# Dual Immune Checkpoint Inhibition Plus Neoadjuvant Chemoradiotherapy in Rectal Cancer: A Randomized Clinical Trial

**DOI:** 10.1001/jamanetworkopen.2025.27769

**Published:** 2025-08-22

**Authors:** Johannes Laengle, Irene Kuehrer, Askin Kulu, Julijan Kabiljo, Daphni Ammon, Rebecca Zirnbauer, Anton Stift, Friedrich Herbst, Bernhard Dauser, Matthias Monschein, Peter Razek, Stefanie Haegele, Matthias Biebl, Hans Geinitz, Wolfgang Hulla, Polina Kalinina, Leonhard Müllauer, Joachim Widder, Clemens Bittermann, Dietmar Pils, Dietmar Tamandl, Friedrich Laengle, Rainer Schmid, Michael Bergmann

**Affiliations:** 1 Division of Visceral Surgery, Department of General Surgery, Comprehensive Cancer Center Vienna, Medical University of Vienna, Vienna, Austria; 2 Department of Surgery, Hospital of St John of God, Vienna, Austria; 3 Department of Surgery, Clinic Floridsdorf, Vienna Hospital Association, Vienna, Austria; 4 Department of Pathology, Clinic Floridsdorf, Vienna Hospital Association, Vienna, Austria; 5 Department of General and Visceral Surgery, Ordensklinikum Linz, Barmherzige Schwestern, Linz, Austria; 6 Faculty of Medicine, Johannes Kepler University Linz, Linz, Austria; 7 Department of Radiation Oncology, Ordensklinikum Linz, Barmherzige Schwestern, Linz, Austria; 8 Institute of Pathology, State Hospital Wiener Neustadt, Neustadt, Austria; 9 Department of Pathology, Comprehensive Cancer Center Vienna, Medical University of Vienna, Vienna, Austria; 10 Department of Radiation Oncology, Comprehensive Cancer Center Vienna, Medical University of Vienna, Vienna, Austria; 11 Clinical Department of General, Visceral and Vascular Surgery, University Hospital Wiener Neustadt, Neustadt, Austria; 12 Department of Biomedical Imaging and Image-guided Therapy, Comprehensive Cancer Center Vienna, Medical University of Vienna, Vienna, Austria

## Abstract

**Importance:**

Immune checkpoint inhibitors (ICIs) show efficacy in treatment of several solid tumors, but microsatellite-stable rectal cancer is largely resistant. Radiotherapy may enhance tumor immunogenicity and thus may make the combination of radiotherapy and ICIs a promising strategy to treat rectal cancer. While anti–programmed cell death protein 1 antibodies in neoadjuvant regimens have been linked to higher complete response rates, the added benefit of including a cytotoxic T-lymphocyte–associated protein 4 (CTLA-4) inhibitor remains unclear.

**Objective:**

To evaluate the safety and feasibility of combining ipilimumab and nivolumab with neoadjuvant chemoradiotherapy (CRT) for rectal cancer.

**Design, Setting, and Participants:**

The CHINOREC trial was a prospective, randomized, open-label, multicenter phase 2 clinical trial conducted from June 2, 2020, to March 15, 2024, across multiple academic and tertiary medical centers in Austria. Analysis was based on intention to treat.

**Intervention:**

Neoadjuvant CRT consisted of 50 Gy in 2-Gy fractions with concurrent capecitabine (1650 mg/m^2^/d). The experimental arm received additional intravenous ipilimumab (1 mg/kg on day 7) and nivolumab (3 mg/kg every 2 weeks starting on day 14) (CRT plus ipilimumab and nivolumab group). Surgical resection was performed 10 to 12 weeks after CRT.

**Main Outcome and Measures:**

The primary outcome was the safety and feasibility of combining CRT with sequential ipilimumab and nivolumab, assessed by surgical complications and reoperation rates. Secondary outcomes included clinical and pathological response rates.

**Results:**

Of the 145 patients assessed, 80 were randomized to receive either CRT alone (CRT group) (n = 30) or to the CRT plus ipilimumab and nivolumab group (n = 50) (49 male [61%]; median age, 60 [range, 36-83] years). Differences in surgical complication rates were not statistically significant between the CRT and CRT plus ipilimumab and nivolumab groups (any grade, 20 of 26 patients [77%] vs 33 of 43 [77%]; *P* > .99), as were reoperation rates (2 of 26 [8%] vs 3 of 43 [7%]; *P* > .99). Major pathological response (10 of 26 [38%] vs 16 of 43 [37%]; *P* > .99) and complete response (9 of 30 [30%] vs 11 of 50 [22%]; *P* = .44) rates were overall high in both groups.

**Conclusions and Relevance:**

In this randomized clinical trial of patients with rectal cancer, integrating ipilimumab and nivolumab into neoadjuvant CRT was safe and feasible, with no increase in surgical complications. Although complete response rates did not significantly improve, the dual ICI regimen demonstrated promising clinical activity. These findings support further translational research to optimize timing, dosing, and fractionation of radiotherapy and ICI therapy and to guide patient selection.

**Trial Registration:**

ClinicalTrials.gov Identifier: NCT04124601

## Key Points

**Question:**

Is dual immune checkpoint inhibition (ICI) with ipilimumab and nivolumab safe and feasible in combination with neoadjuvant chemoradiotherapy (CRT) for rectal cancer?

**Findings:**

In this randomized clinical trial of 80 patients with rectal cancer, the differences between the CRT and the CRT plus ICI groups in the proportion of surgical complications (77% vs 77%, respectively) and reoperation rates (8% vs 7%, respectively) were not statistically significant.

**Meaning:**

Integrating ipilimumab and nivolumab into neoadjuvant CRT is safe and feasible, warranting further translational analyses to identify the optimal timing and dosing of radiotherapy and ICIs.

## Teaser

This randomized clinical trial evaluates the safety of sequentially administered neoadjuvant ipilimumab and nivolumab with concurrent chemoradiotherapy in patients with rectal cancer scheduled for surgical resection.

## Introduction

Immune checkpoint inhibitors (ICIs) have been proven effective in various solid cancers by restoring the immune system’s ability to target cancer cells, leading to improved cure rates.^1,2^ However, microsatellite-stable (MSS) colorectal cancer generally remains resistant to ICIs, despite cytotoxic T lymphocytes (CTLs) being key prognostic factors.^3,4^ In contrast, patients with microsatellite-instable colorectal cancer (10%-15%) respond favorably to ICIs,^3,5,6^ suggesting that MSS colorectal cancer could be made responsive to ICIs if the tumor immune microenvironment gets appropriately primed.^7,8^

Stary et al^9^ previously demonstrated that radiotherapy can induce a proinflammatory phenotype in tumor-infiltrating macrophages, highlighting radiotherapy’s potential role in activating an effective tumor immune microenvironment. Combinations of radiotherapy and ICIs are currently under investigation in various cancers, as preclinical studies indicate promising synergy.^10,11^ Neoadjuvant ICIs, alone or with chemotherapy, have shown significant benefits, including enhanced recurrence-free survival.^12,13,14,15,16,17,18,19,20^ This innovative approach has the potential to change clinical practice, prompting rapid expansion to a range of tumor types and stages.^21,22,23,24,25,26^

In rectal cancer, recent studies suggested that anti–programmed cell death protein 1 (PD-1) monotherapy, when combined with different neoadjuvant treatment regimens, increased the complete response rate.^25,26,27^ However, the potential benefit of adding anti–cytotoxic T lymphocyte–associated protein 4 (CTLA-4) antibodies remains unclear. Preclinical evidence suggested that anti–CTLA-4 antibodies deplete regulatory T cells and enhance T-cell priming, while anti–PD-1 antibodies reduce T-cell exhaustion.^28,29,30,31,32^ In turn, these findings suggest that a sequential blockade of CTLA-4 followed by PD-1 might provide an added benefit by optimally priming the immune response, preventing T-cell dysfunction and resistance to anti–PD-1 therapy.^33^

In this randomized clinical trial, we evaluated the safety of sequentially administered neoadjuvant ipilimumab and nivolumab with concurrent chemoradiotherapy (CRT) in patients with rectal cancer scheduled for surgical resection. Careful consideration of ICI-induced immune-related adverse events (irAEs) is crucial, as they may necessitate immunosuppressive therapy.^34,35,36^ Therefore, our primary end point was surgical safety, given that surgery remains the mainstay for achieving cure in most patients with rectal cancer, even in the evolving era of “watch and wait.”

## Methods

### Study Design and Participants

The CHINOREC study is a prospective, randomized, open-label, Austrian multicenter, phase 2 investigator-initiated clinical trial.^37,38,39^ The trial protocol is found in Supplement 1. Patients with rectal cancer were randomized to receive either neoadjuvant CRT alone (CRT group) or with sequential ipilimumab (single dose) and nivolumab (3 cycles) (CRT plus ipilimumab and nivolumab group). Patients were scheduled for curative surgical resection 10 to 12 weeks after CRT (eFigure 1 in Supplement 2).

Key inclusion criteria were older than 18 years, histologically confirmed rectal carcinoma, and the medical indication for neoadjuvant CRT.^40^ Eligible stages included cT3a to cT3b in the low rectum (mesorectal fascia negative) or middle to high rectum; cN1 to cN2, negative for extramural vascular invasion (EMVI); or cT3b positive for EMVI. Major exclusion criteria included incurable metastatic disease and any contraindications to CRT or ICI therapy based on a summary of product characteristics. Full eligibility criteria are provided in the online protocol (Supplement 1). Ethnicity data were classified by physicians using predefined options (from principal investigators [J.L. and M.B.]) to assess health disparities, treatment responses, and disease prevalence.

The study protocol was approved by the lead ethics committee of the Medical University of Vienna and endorsed by the local ethics committee of each participating center. The study adhered to the International Council for Harmonisation guidelines for Good Clinical Practice, the Declaration of Helsinki,^41^ and Austrian health regulations. Written informed consent was obtained from all participants prior to randomization. This study was reported according to the Consolidated Standards of Reporting Trials (CONSORT) reporting guidelines.

### Randomization and Masking

Patients were randomized (30:50) using a permuted block design (block size = 8) via Randomizer for Multicenter Trials (Medical University of Graz). This ratio was selected to maximize data collection in the experimental arm while ensuring a sufficiently sized control group for safety validation, without exceeding feasibility constraints for patient recruitment. It closely approximates a 1:2 ratio but with rounded numbers for practicality. The open-label design did not require any masking.

### Procedures

External beam radiotherapy was performed using volumetric-modulated arc therapy to deliver a total of 50 Gy in 2-Gy fractions (Monday to Friday) with concurrent capecitabine (1650 mg/m^2^/d). Cone beam computed tomography was used for image guidance. Intravenous ipilimumab (1 mg/kg for 30 minutes) was administered on day 7 after CRT initiation, followed by intravenous nivolumab (3 mg/kg for 30 minutes) every 2 weeks for 3 doses starting on day 14. Surgical procedures included total mesorectal excision, intersphincteric resection, or abdominoperineal resection performed laparoscopically, robotically, or via an open approach 10 to 12 weeks after CRT.

### Outcomes

The primary outcome was to assess the safety of adding ipilimumab and nivolumab concurrently to neoadjuvant CRT, followed by surgical resection, compared with CRT alone. This was evaluated using the Clavien-Dindo Classification, version 2.0, and the Common Terminology Criteria for Adverse Events, version 5.0.

Key secondary outcome consisted of the pathological and clinical response rates. Pathological staging was compliant with the *American Joint Committee on Cancer Staging Manual*, eighth edition. Pathological response was classified according to the Dworak tumor regression grade (TRG 0-4).^42^ TRG 4 is equivalent to a pathological complete response and TRG 3 to a near-pathological complete response. A major pathological response (MPR) was defined as 10% or less residual vital tumor, equivalent to TRG 3 to 4. Consequently, a non-MPR was defined as TRG 0 to 2. A pathological partial response was defined as 50% or less residual vital tumor, equivalent to TRG 2. Assessment of clinical response used rectal magnetic resonance imaging with diffusion-weighted imaging, endoscopy, carcinoembryonic antigen level, and digital rectal examination. Clinical response was classified according to the magnetic resonance TRG (mrTRG 1-5).^43^ A major clinical response was defined as mrTRG 1 to 2, whereas a nonmajor clinical response was defined as mrTRG 3 to 5. A clinical partial response was defined as mrTRG 3. Clinical response assessments were independently reviewed by an expert gastrointestinal tract radiologist (D.T.). Additionally, the neoadjuvant rectal score was calculated using the following equation: [5*ypN* – 3(*cT* – *ypT*) + 12]^2^/9.61. Responses were subgrouped into low (<8 [excellent]), intermediate (8-16), or high (>16 [poor]).^44,45^

### Analysis of Mismatch Repair Status

Formalin-fixed paraffin-embedded tumor samples were analyzed for mismatch repair (MMR) status using immunohistochemistry to detect *MLH1*, *MSH2*, *MSH6*, and *PMS2* (eTable 1 in Supplement 2) on an immunostainer (VENTANA BenchMark ULTRA; Department of Pathology, Medical University of Vienna). Deficient MMR was characterized by protein loss, with matched noncancerous tissue used as controls. Analysis was performed on pretreatment biopsy specimens when sufficient tissue was available; otherwise, the surgical resection specimen was used.

### Tumor Mutation Analysis

Next-generation sequencing of formalin-fixed paraffin-embedded tumor tissues was conducted using the Ion GeneStudio S5 System (Thermo Fisher Scientific Inc) with the Ion AmpliSeq Colon and Lung Cancer Research Panel v2 (Department of Pathology, Medical University of Vienna). Data were analyzed with Ion Reporter Software, version 5.20 (Thermo Fisher Scientific Inc). Tumor mutational analysis was primarily performed on surgical specimens rather than biopsy specimens due to limited tissue availability.

### Statistical Analysis

Analysis was based on intention to treat. Given the absence of published data on neoadjuvant ICI combined with CRT in curative-intent rectal cancer, the study was designed to demonstrate that adding ipilimumab and nivolumab to standard CRT does not lead to an unacceptable increase in surgical morbidity rather than to power oncologic efficacy outcomes. An informal power analysis, based on published surgical complication rates in patients undergoing neoadjuvant CRT for rectal cancer, indicated that with 49 patients in the CRT plus ipilimumab and nivolumab arm, the study would have 90% power (α = .05) to exclude a doubling (100% increase) of the expected rate of any surgical complications (23%).^46^

Safety (primary outcome) was assessed by comparing the observed rate of any postoperative complications in the CRT plus ipilimumab and nivolumab arm with the calculated upper 95% CI of the expected rate (39%).^46^ The CRT arm served to validate these expected rates from historical data. Expected case numbers and their corresponding 95% CIs are detailed in the study protocol (Supplement 1). This approach aligns with the principles of a noninferiority analysis, where exceeding the predefined 95% CI boundary would indicate that the treatment is unsafe, although the study was not formally structured as a noninferiority trial. Interim analyses were conducted after every 10th patient in the CRT plus ipilimumab and nivolumab arm to monitor reoperation rates against historical benchmarks.^46^ The study was set to terminate if the upper 95% CI for reoperation rates was exceeded at any interim analysis. The predefined cutoff values for termination are detailed in the study protocol (Supplement 1).

All statistical analyses were performed using R, version 4.4.1 (R Program for Statistical Computing).^47^ The stats package, version 3.6.0, and the binom.test function were used to calculate the 95% CI for postoperative complications. Rounding of case numbers was performed using a round half up method, instead of the default round to even function in R. Fisher exact test (for 2 × 2 tables) or χ^2^ test (for >2 categories) was applied to compare frequency distributions between the 2 groups; the Mann-Whitney test was used for continuous data. Statistical significance was defined as 2-sided *P* < .05.

## Results

Between June 2, 2020, and March 15, 2024, 145 patients were screened for eligibility, and 80 were randomized to receive either CRT alone (n = 30) or CRT combined with ipilimumab and nivolumab (n = 50) (31 female [39%] and 49 male [61%] patients; median age, 60 [range, 36-83] years; 79 [99%] European and 1 [1%] Filipino). Baseline characteristics were well balanced between groups, with no significant differences observed except for a modest difference in median body mass index (25 [range, 21-32] vs 26 [range, 18-43]; *P* = .02 [calculated as the weight in kilograms divided by the height in square meters]), which was not deemed clinically meaningful (Table 1). The median age was 62 (range, 36-78) years in the CRT arm and 60 (range, 44-83) years in the CRT plus ipilimumab and nivolumab arm. The distribution of male and female patients was not statistically different, with males representing 18 (60%) in the CRT arm and 31 (62%) in the CRT plus ipilimumab and nivolumab arm and females representing 12 (40%) and 19 (38%), respectively. Tumor location was also not statistically different between groups (*P* = .06), although a numerical difference was observed in the proportion of tumors located in the lower rectum (12 [40%] in the CRT arm vs 27 [54%] in the CRT plus ipilimumab and nivolumab arm). Tumors staged as cT4 were present in both groups at similar rates (4 [13%] in the CRT arm vs 6 [12%] in the CRT plus ipilimumab and nivolumab arm). The overall distribution of magnetic resonance EMVI status was not statistically different between groups (*P* = .21); however, a higher proportion of positive magnetic resonance EMVI was observed in the CRT group (7 [23%] vs 5 [10%]).

**Table 1.** Baseline Patient Characteristics of Intention-to-Treat Population

Characteristic	Patient group		*P* value
	CRT (n = 30)	CRT plus IPI-NIVO (n = 50)	
Age, median (range), y	62 (36-78)	60 (44-83)	.98
Sex, No. (%)			
Female	12 (40)	19 (38)	>.99
Male	18 (60)	31 (62)	
Ethnicity, No. (%)			
European	29 (97)	50 (100)	.38
Filipino	1 (3)	0	
ECOG 0-1, No. (%)	30 (100)	50 (100)	>.99
BMI, median (range)	25 (21-32)	26 (18-43)	.02
Tumor location from dentate line, No. (%)			
Upper (>12-16 cm)	3 (10)	0	.06
Middle (6-12 cm)	15 (50)	23 (46)	
Lower (<6 cm)	12 (40)	27 (54)	
cT stage, No. (%)			
cT2	2 (7)	2 (4)	.85
cT3	24 (80)	42 (84)	
cT4	4 (13)	6 (12)	
cN stage, No. (%)			
cN0	3 (10)	3 (6)	.67
cN+	27 (90)	47 (94)	
cM0 stage, No. (%)	30 (100)	50 (100)	>.99
mrMRF status, No. (%)			
Negative (>1 mm)	18 (60)	21 (42)	.24
Endangered	4 (13)	13 (26)	
Positive (≤1 mm)	8 (27)	16 (32)	
mrEMVI status, No. (%)			
Negative	20 (67)	36 (72)	.21
Endangered	3 (10)	9 (18)	
Positive	7 (23)	5 (10)	

Abbreviations: BMI, body mass index (calculated as the weight in kilograms divided by the height in square meters); CRT, chemoradiotherapy; ECOG, Eastern Cooperative Oncology Group; IPI, ipilimumab; mrMRF, magnetic resonance mesorectal fascia; mrEMVI, magnetic resonance extramural vascular invasion; NIVO, nivolumab.

In the CRT arm, all 30 patients received the planned full course of CRT. In the CRT plus ipilimumab and nivolumab arm, all 50 patients completed the CRT regimen and received the scheduled dose of ipilimumab. The CONSORT flow diagram is provided in eFigure 2 in Supplement 2. Nivolumab was administered sequentially, with the first dose given to 49 patients (98%), the second dose to 46 patients (92%), and the third dose to 40 patients (80%). Reasons for discontinuation of nivolumab therapy included hepatitis (n = 5), myositis (n = 5), diarrhea (n = 3), thyroiditis (n = 1) and COVID-19 infection (n = 1). Surgical resection was completed in 69 patients (86%) in both groups (26 of 30 in the CRT arm and 43 of 50 in the CRT plus ipilimumab and nivolumab arm). In the CRT arm, 4 patients did not undergo surgery due to disease progression (n = 1) or patient choice (n = 3). In the CRT plus ipilimumab and nivolumab group, 7 patients did not proceed to surgery due to disease progression (n = 1), poor performance status following a trauma (n = 1), and patient choice (n = 5). Patient preference was based on a suspected clinical complete response or near-clinical complete response, leading to a decision to pursue organ-preserving nonoperative management, according to a watch-and-wait strategy.

Among patients who underwent tumor resection, minimally invasive surgery was used in 54 of 69 cases (78%). Differences in the distribution of surgical approaches (open, laparoscopic, or robotic) were not statistically significant between the CRT and CRT plus ipilimumab and nivolumab groups. The proportion of open procedures, including conversions from laparoscopic or robotic approaches, displayed some variance (8 [31%] in the CRT arm vs 7 [16%] in the CRT plus ipilimumab and nivolumab arm). Surgical characteristics are shown in Table 2.

**Table 2.** Surgical Characteristics

Characteristic	Patient group	
	CRT (n = 30)	CRT plus IPI-NIVO (n = 50)
Received surgery, No. (%)		
Yes	26 (87)	43 (86)
No^a^	4 (13)	7 (14)
Type of resection, No. (%)		
Total mesorectal excision	23 (88)	39 (91)
Intersphincteric resection	1 (4)	0
Abdominoperineal resection	2 (8)	4 (9)
Surgical approach, No. (%)		
Laparoscopic	9 (35)	12 (28)
Robotic	9 (35)	24 (56)
Open^b^	8 (31)	7 (16)
Protective loop ileostomy, No. (%)		
Yes	24 (92)	38 (88)
No	2 (8)	5 (12)

Abbreviations: CRT, chemoradiotherapy; IPI, ipilimumab; NIVO, nivolumab.

^a^ Owing to patient’s choice (clinical or near-clinical complete response), clinical progressive disease, or poor Eastern Cooperative Oncology Group status.

^b^ Including conversion from laparoscopic or robotic.

**Figure 1.**
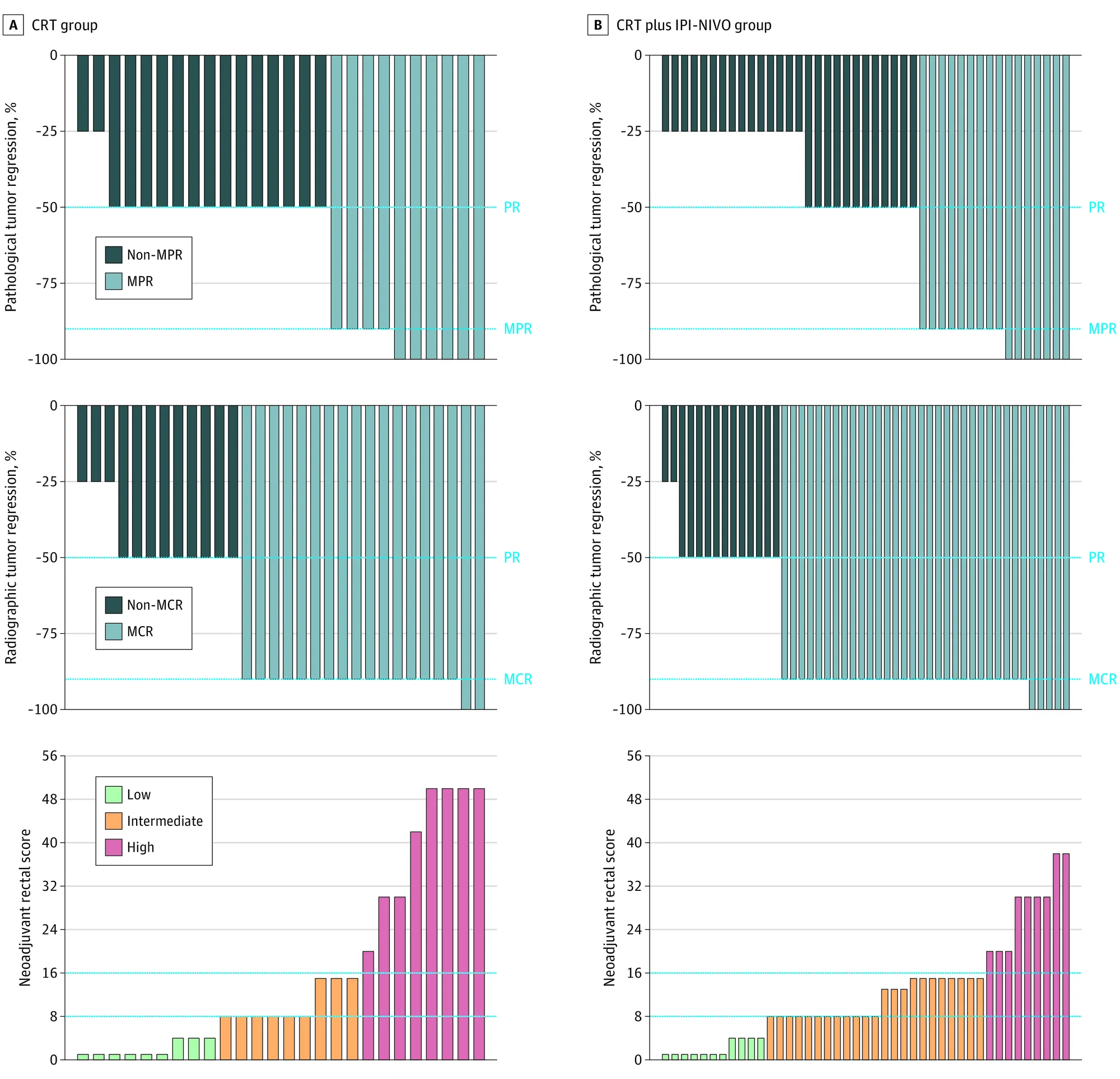
Clinical and Pathological Response and Neoadjuvant Rectal Score Percentages of pathological tumor regression (TRG) and radiographic tumor regression (magnetic resonance TRG [mrTRG]), along with neoadjuvant rectal scores for each individual patient are shown. The blue lines indicate thresholds for partial response (PR), major clinical response (MCR), major pathological response (MPR), and neoadjuvant rectal score categories (low, intermediate and high). CRT indicates chemoradiotherapy; IPI, ipilimumab; and NIVO, nivolumab.

The incidence of the primary end point, any surgical complication, was 20 of 26 patients (77%) in the CRT group and 33 of 43 (77%) in the CRT plus ipilimumab and nivolumab group (*P* > .99) (Figure 1 and eTable 1 in the Supplement 2). Correspondingly, reoperation rates (Clavien-Dindo classification grade ≥IIIb) were comparably low in both groups (2 of 26 [8%] in CRT vs 3 of 43 [7%] in CRT plus ipilimumab and nivolumab; *P* > .99) and did not exceed the prespecified cutoff values in any interim analysis. There was no difference in 90-day postoperative mortality, which occurred in 1 patient (2%) in the CRT plus ipilimumab and nivolumab group due to an ischemic descending colon, complicated by an anastomotic leak, peritonitis, septic shock, and subsequent multiorgan failure, which was unrelated to ipilimumab and nivolumab treatment. Grade IIIa complications (rectal anastomotic leaks) were managed endoscopically with vacuum therapy, avoiding general anesthesia.

**Figure 2.**
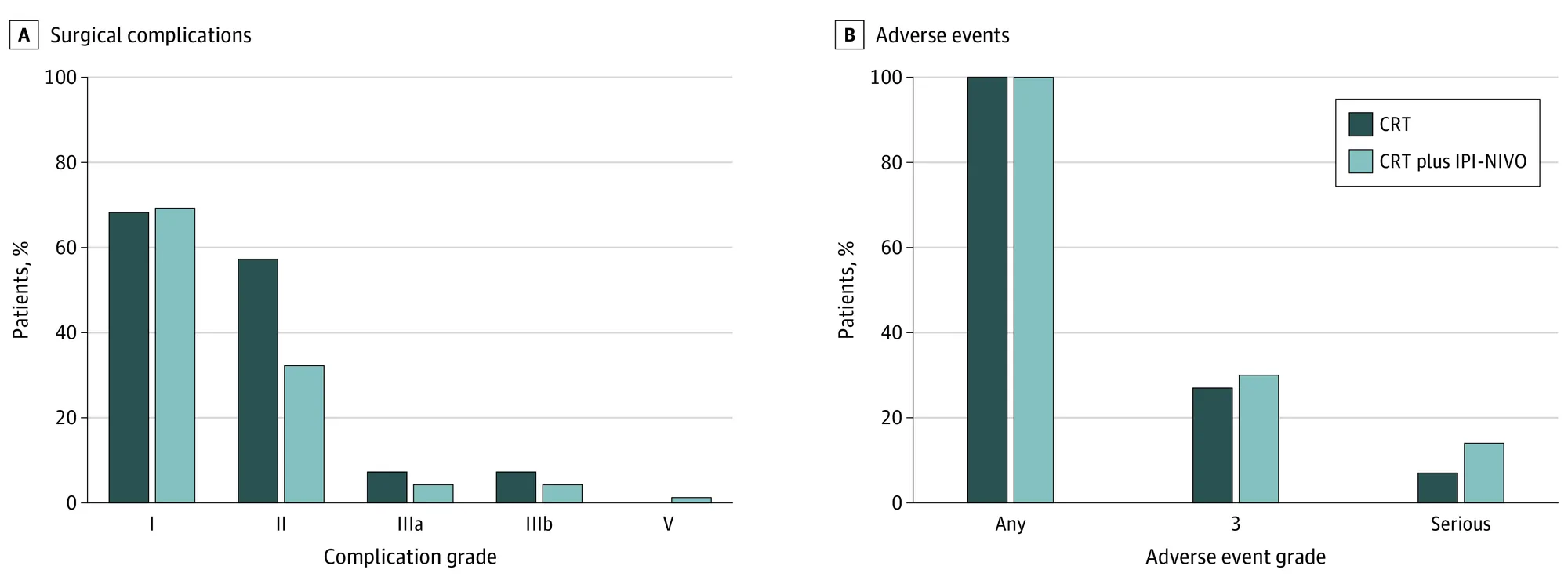
Surgical Complications and Adverse Events Percentages of surgical complications (assessed using Clavien-Dindo Classification, version, 2.0) and adverse events (assessed using Common Terminology Criteria for Adverse Events, version 5.0). CRT indicates chemoradiotherapy; IPI, ipilimumab; and NIVO, nivolumab.

All patients experienced at least 1 adverse event (AE). AEs of grade 3 or greater occurred in 8 of 30 patients (27%) in the CRT arm and 15 of 50 (30%) in the CRT plus ipilimumab and nivolumab arm (*P* = .80), indicating a difference in incidence that was not statistically significant (Figure 2 and eTable 2 in the Supplement 1). Similarly, serious AEs (SAEs) occurred in 7 of 50 patients (14%) in the CRT plus ipilimumab and nivolumab group and 2 of 30 (7%) in the CRT group (*P* = .47). Among the SAEs in the CRT plus ipilimumab and nivolumab arm, those in 3 patients (6%) were directly attributable to the ICI therapy (ie, hepatitis, myositis, and diarrhea), which led to discontinuation of treatment. Detailed distributions of AEs occurring in at least 8 patients (10%) as well as AEs of grade 3 or greater are shown in eFigures 3 and 4 in Supplement 2.

**Figure 3.**
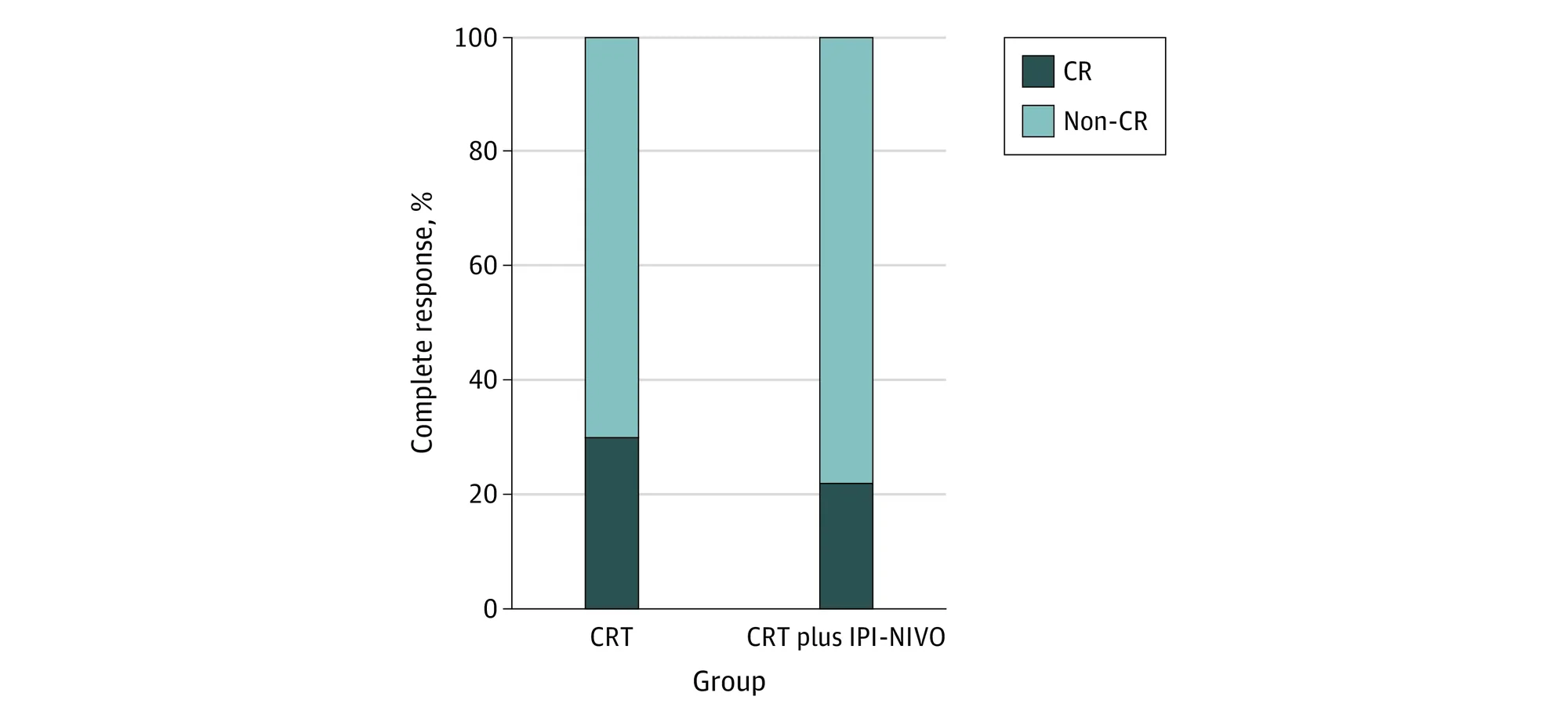
Complete Response Rate Percentages of complete response (clinical or pathological) with treatment groups. CR indicates complete response; CRT, chemoradiotherapy; IPI, ipilimumab; and NIVO, nivolumab.

The key secondary end point of complete response rate (clinical or pathological) was 9 of 30 (30%) in the CRT group and 11 of 50 (22%) in the CRT plus ipilimumab and nivolumab group (*P* = .44) (Figure 3 and eTable 3 in Supplement 2). The rate of major clinical response occurred in 34 of 48 patients (71%) in the CRT plus ipilimumab and nivolumab group and 18 of 30 (60%) in the CRT group (*P* = .34). MPR rates were 10 of 26 patients (38%) in the CRT arm and 16 of 43 (37%) in the CRT plus ipilimumab and nivolumab arm (*P* > .99), with no notable differences observed between groups. Likewise, the distribution of neoadjuvant rectal scores appeared not statistically different between groups (eg, intermediate, 9 of 26 [35%] vs 23 of 43 [53%]; *P* = .31) (Figure 3 and eTable 3 in Supplement 2).

Results of histopathological examination (eTable 4 in Supplement 2) showed that the distribution of tumor differentiation grades did not differ statistically between the CRT and CRT plus ipilimumab and nivolumab groups. Differences in rates of advanced ypT and ypN stages remained statistically insignificant between the 2 groups, with both achieving high rates of ypN0 status (31 of 43 [72%] in the CRT plus ipilimumab and nivolumab arm vs 18 of 26 [69%] in the CRT arm; *P* = .30). Molecular analyses revealed that both treatment arms had a high prevalence of proficient MMR status, with no cases of deficient MMR in the CRT plus ipilimumab and nivolumab arm. The rates of mutations in key oncogenes, such as *KRAS*, *TP53,* or *PIK3CA*, were not statistically different between the groups, indicating that the observed differences in response rates were likely driven by treatment effects rather than intrinsic tumor genetics (eTable 4 in Supplement 2).

## Discussion

In this phase 2 randomized clinical trial, we evaluated the safety of adding dual ICIs, specifically ipilimumab and nivolumab, to neoadjuvant CRT in patients with rectal cancer. Our findings demonstrated that integrating ipilimumab and nivolumab treatment with CRT was both safe and feasible, with differences in surgical outcomes and complication rates not statistically significant compared with those of CRT alone. Notably, this combination therapy achieved an MPR rate of 37% and a complete response rate of 22%.

The primary end point was to assess surgical safety and feasibility, given the critical role of surgical resection in curative-intent treatment for rectal cancer. We observed reoperation rates of 7% to 8% in both treatment arms, aligning with the benchmark set by the Stockholm III trial (7%).^46^ These findings indicate that adding a CTLA-4 inhibitor to an anti−PD-1 inhibitor concurrently with neoadjuvant CRT does not exacerbate complications in surgical resections. We believe this is clinically relevant, as CTLA-4 inhibitors are generally associated with increased irAEs. Specifically, patients receiving anti–PD-1 monotherapy experienced irAEs at a rate of 74%, with 14% experiencing events of grade 3 or greater.^48^ In contrast, combining PD-1 with CTLA-4 inhibition led to a higher irAE incidence (90%), with 55% of patients experiencing AEs of grade 3 or greater.^48^

In our study, the incidence of AEs of grade 3 or greater and SAEs in the CRT plus ipilimumab and nivolumab arm were 30% and 14%, respectively, with only 6% directly attributable to ICIs. These results were consistent with previously published data on anti–PD-1 monotherapies combined with various neoadjuvant regimens.^25,26,27^ However, 20% of patients in the CRT plus ipilimumab and nivolumab group required discontinuation due to ICI-induced hepatitis, myositis, or diarrhea. This underscores the need for vigilant monitoring and prompt intervention, which are crucial when using ICIs in curative settings. Immunosuppressive therapy use is challenging in bowel surgery with delicate anastomoses, and ICI-induced thyroiditis requiring lifelong hormone therapy must be considered. The use of a limited ICI dosing schedule (1 dose of ipilimumab and 3 doses of nivolumab) aligns with emerging neoadjuvant trial designs in curative-intent settings, where short-course ICI therapy is favored to minimize irAEs and avoid perioperative complications, in contrast to prolonged ICI regimens used in metastatic disease.

Regarding the secondary end point, our analysis did not reveal significant differences in complete response rates between the 2 arms (30% for CRT vs 22% for CRT plus ipilimumab and nivolumab). Conventional neoadjuvant CRT typically achieves pathological complete response rates of approximately 14%.^49^ However, studies by Habr-Gama et al^50,51^ have reported higher rates (26% pathological complete response and 33% clinical complete response with organ preservation). Our overall complete response rates, however, were comparable with those in recent trials for total neoadjuvant therapy (TNT), such as PRODIGE 23 (Partenariat de Recherche en Oncologie Digestive; pathological complete response rate, 27%),^52^ RAPIDO (Rectal Cancer and Preoperative Induction Therapy Followed by Dedicated Operation; pathological complete response rate, 28%),^53^ and STELLAR (pathological complete response rate, 21%).^54^ However, the SAE rates in these TNT regimens were considerably higher (27% for PRODIGE 23 and 38% for RAPIDO),^52,53^ whereas our CRT plus ipilimumab and nivolumab arm had a lower incidence of ICI-related SAEs (6%). The validity of surrogate end points such as complete response for long-term overall survival remains to be elucidated over time, as, for example, the initial promising pathological complete response of the RAPIDO trial resulted in 12% locoregional failure.^55^ In addition, our decision to schedule surgery at 10 to 12 weeks after CRT was based on recent international consensus guidelines, which recommend assessing clinical response at 12 weeks from treatment initiation, with later reassessment for patients with near-complete response.^56^ This approach, adopted in trials such as STAR-TREC^57^ and OPERA,^58^ allows for maximal tumor regression and more accurate identification of patients with potential complete response. Compared with TNT regimens involving extended induction or consolidation chemotherapy, the total neoadjuvant treatment duration in our study was shorter. This difference may partly account for the observed variability in pathological response rates across studies.

Recent randomized clinical trials integrating anti–PD-1 monotherapies into various TNT regimens have shown stimulating results. For example, the TORCH trial (short-course radiotherapy following 6 cycles of CAPOX [capecitabine plus oxaliplatin] plus anti–PD-1)^25^ reported a pathological complete response rate of greater than 50%, the UNION trial (short-course radiotherapy following 2 cycles of CAPOX plus anti–PD-1)^26^ achieved a pathological complete response rate of 39%, Xiao et al^27^ (4 × CAPOX plus CRT plus anti–PD-1) reported a total complete response rate of 44%, and the NRG-GI002 study (6 cycles FOLFOX [folinic acid, fluorouracil, and oxaliplatin] following CRT plus 6 cycles of anti–PD-1)^59^ reported a pathological complete response rate of 31%. Single-arm studies such as VOLTAGE-A (CRT following 5 cycles of anti–PD-1)^60^ and one by Lin et al^61^ (short-course radiotherapy plus 2 cycles of CAPOX plus anti–PD-1) reported pathological complete response rates of 30% and 46%, respectively. Although direct comparisons of those randomized studies are lacking, the complete response data from our trial indicate that adding oxaliplatin to a neoadjuvant CRT regimen for rectal cancer may specifically enhance the efficacy of ICIs in achieving a complete response. These findings underscore the complexity of integrating ICIs into neoadjuvant strategies for proficient MMR–MSS rectal cancer. As TNT has become the new standard of care for locally advanced rectal cancer, our findings support further investigation of whether dual ICIs can be integrated into or serve as an alternative to existing TNT regimens, particularly for patients unable to tolerate intensive chemotherapy.

### Limitations

Our study has several limitations. First, the sample size was adequate for safety assessment but may have lacked power for subtle efficacy differences. Second, the open-label design could introduce bias in subjective end points such as complete response rates. Third, interim analyses may have influenced the nominal coverage of the 95% CIs, although enrollment was completed without early termination, supporting safety conclusions. Last, the study had a lack of long-term efficacy end points such as recurrence-free survival and overall survival, which were not prespecified or powered within this phase 2 design. While pathological response metrics such as pathological complete response and MPR are widely accepted surrogate markers in early-phase trials, they may not fully capture the long-term clinical benefit.

## Conclusions

In this randomized clinical trial, adding ipilimumab and nivolumab to neoadjuvant CRT in rectal cancer is safe, with no increase in surgical complications or SAEs. However, it did not significantly improve complete response rates over CRT alone, indicating that dual ICIs may need further refinement for clinical benefit in proficient MMR–MSS rectal cancer. Further research is needed to optimize ICI combinations, sequencing, and dosing with radiotherapy in the neoadjuvant setting. Biomarker studies could identify patients most likely to benefit, while larger trials with longer follow-up are essential to assess long-term survival and quality of life.

## Notes

Supplement 1.
Trial Protocol

Supplement 2. **eTable 1.** Surgical Morbidity **eTable 2.** Summary of Adverse Events (Treatment-Related or Not) **eTable 3.** Therapy Response **eTable 4.** Pathological Characteristics **eFigure 1.** Study Design **eFigure 2.** Consort Flow Diagram **eFigure 3.** Distribution of AEs Occurring in at Least 10% of All Patients **eFigure 4.** Distribution of AE Grades ≥3

Supplement 3.
Data Sharing Statement
