# Error in Figure 1

**DOI:** 10.1001/jamanetworkopen.2026.41974

**Published:** 2026-10-05

In the Original Investigation titled, “Dual Immune Checkpoint Inhibition Plus Neoadjuvant Chemoradiotherapy in Rectal Cancer: A Randomized Clinical Trial,”^1^ published August 22, 2025, there was an error in the color of the bars in the bottom panel of Figure 1B. The bars at the right with a neoadjuvant score above 16 were changed from green to red, indicating a high score. This article has been corrected.^1^
