# Supplementary material for: Dual Immune Checkpoint Inhibition Plus Neoadjuvant Chemoradiotherapy in Rectal Cancer: A Randomized Clinical Trial
Source: JAMA Netw Open. 2025 Aug 22;8(8):e2527769. doi: 10.1001/jamanetworkopen.2025.27769 (PMC12374221; doi:10.1001/jamanetworkopen.2025.27769)
Supplement: Supplement 1. — Trial Protocol [file jamanetwopen-e2527769-s001.pdf]

Clinical Study Protocol

Neoadjuvant CHemoradiotherapy with sequential Ipilimumab and NivOlumab in RECtal cancer (CHINOREC): a prospective randomized, open-label, multicenter, phase II clinical trial

CHINOREC

1.10 / 15.01.2022

Confidentiality Statement

The information contained in this document, especially unpublished data, is the property of the sponsor of this study. It is therefore provided to you in confidence as an Investigator, potential Investigator, or consultant, for review by you, your staff, and an Independent Ethics Committee or Institutional Review Board. It is understood that this information will not be disclosed to others without written authorization from the investigators, except to the extent necessary to obtain informed consent from those persons to whom the study drug may be administered.

**Test drug (IMP) and Pharmaceutical Company**	Ipilimumab (Yervoy®), Nivolumab (Opdivo®); Bristol-Myers Squibb (BMS)
**Protocol authors**	Dr.med.univ. Johannes Längle, PhD<br>Ao.Univ.-Prof. Dr.med.univ. Michael Bergmann
**Principal-Investigator**<br>**Sub-Investigator**	Ao.Univ.-Prof. Dr.med.univ. Michael Bergmann<br>Dr.med.univ. Johannes Längle, PhD
**Document type**	Clinical study protocol
**Study phase**	II
**Document status**	Final draft
**Date**	15.01.2022
**Number of pages**	49

1 SPONSOR, INVESTIGATOR, MONITOR AND SIGNATURES

Sponsor

Medical University of Vienna

Univ.-Prof. Priv.-Doz. Dr. Oliver Strobel, MBA

Representative of the dean as the head of Department of General Surgery and Division of Visceral Surgery, Comprehensive Cancer Center Vienna, Medical University of Vienna, Austria

Signature (OEL)	Date

Principal-Investigator (AMG §§ 2a, 35, 36)

Ao.Univ.-Prof. Dr.med.univ. Michael Bergmann

Division of Visceral Surgery, Department of General Surgery, Comprehensive Cancer Center Vienna, Medical University of Vienna, Austria

Signature	Date

Sub-Investigator (AMG §§ 2a, 35, 36)

Dr.med.univ. Johannes Längle, PhD

Division of Visceral Surgery, Department of General Surgery, Comprehensive Cancer Center Vienna, Medical University of Vienna, Austria

Signature	Date

Monitor/ or Representative of CRO (AMG §§ 2a, 33, 34)

Univ.-Prof. Dr.med.univ. Michael Wolzt

Clinical Trials Coordination Centre, Department of Clinical Pharmacology, Medical University of Vienna, Austria

Signature	Date

Statistician

Priv.-Doz. Mag. Dr. Dietmar Pils, MSc, MA

Institute of Clinical Biometrics, Center for Medical Statistics, Informatics and Intelligent Systems, Medical University of Vienna, Vienna, Austria

Division of Visceral Surgery, Department of General Surgery, Comprehensive Cancer Center Vienna, Medical University of Vienna, Austria

Signature

Date

Radiation Oncologist

Dr.med.univ. Rainer Schmid

Department of Radiation Oncology, Comprehensive Cancer Center Vienna, Medical University of Vienna, Austria

Clinical Oncologist

Ass.-Prof. Dr.med.univ. Irene Kührer

Department of Internal Medicine III, Comprehensive Cancer Center Vienna, Medical University of Vienna, Austria

Clinical Trials Centers

Vienna General Hospital, Medical University of Vienna, Vienna, Austria

State Hospital Wiener Neustadt, Lower Austria, Austria

Hospital of St. John of God, Vienna, Austria

Hospital North - Clinic Floridsdorf, Vienna, Austria

Congregational Hospital Linz - Sisters of Mercy, Linz, Austria

Associated Departments

Division of General Surgery, Comprehensive Cancer Center Vienna, Department of Surgery, Medical University of Vienna, Austria

Department of Pathology, Comprehensive Cancer Center Vienna, Medical University of Vienna, Austria

Division of General and Pediatric Radiology, Comprehensive Cancer Center Vienna, Department of Biomedical Imaging and Image-guided Therapy, Medical University of Vienna, Austria

Institute of Clinical Biometrics, Center for Medical Statistics, Informatics and Intelligent Systems, Medical University of Vienna

Department of Radiotherapy, Comprehensive Cancer Center Vienna, Medical University of Vienna, Austria

Department of Laboratory Medicine, Medical University of Vienna, Austria

Department of Clinical Pharmacology, Medical University of Vienna, Austria

Clinical Trials Coordination Centre, Medical University of Vienna, Austria

Department of Surgery, State Hospital Wiener Neustadt, Lower Austria, Austria

Department of Radiology, State Hospital Wiener Neustadt, Lower Austria, Austria

Department of Pathology, State Hospital Wiener Neustadt, Lower Austria, Austria

Department of Laboratory Medicine, State Hospital Wiener Neustadt, Lower Austria, Austria

Department of General and Visceral Surgery, Congregational Hospital Linz, Sisters of Mercy, Linz, Austria

Department of Internal Medicine I, Congregational Hospital Linz, Sisters of Mercy, Linz, Austria

Department of Radiation Oncology, Congregational Hospital Linz, Sisters of Mercy, Linz, Austria

Department of Pathology, Congregational Hospital Linz, Sisters of Mercy, Linz, Austria

Department of Radiology, Congregational Hospital Linz, Sisters of Mercy, Linz, Austria

Department of Laboratory Medicine, Congregational Hospital Linz, Sisters of Mercy, Linz, Austria

2 PROTOCOL SYNOPSIS

<table>
<tr><td>TITLE</td><td colspan="6">Neoadjuvant CHemoradiotherapy with sequential Ipilimumab and NivOlumab in RECtal cancer (CHINOREC): a prospective randomized, open-label, multicenter, phase II clinical trial</td></tr>
<tr><td>OBJECTIVES</td><td colspan="6">Primary Objective
Safety, tolerability and feasibility assessed by the latest “Clavien-Dindo Classification of surgical complications” and Common Terminology Criteria of Adverse Events (CTCAE).
Secondary Objectives
Radiographic response determined by magnetic resonance imaging-assessed tumor regression grade (mrTRG)
Pathological response determined by tumor regression grade (TRG) and tumor stage (ypT and ypN stage).
Correlate genomic information (i.e. MSI, KRAS, BRAF, TP53, PTEN, PIK3CA, TMB, etc.) with therapy response
Correlate genomic, transcriptomic, epigenomic and/or proteomic pattern of tumor biopsies on day -21 to -1, 14 and 28 with therapy response and clinical outcome.
Correlate genomic, transcriptomic, epigenomic and/or proteomic pattern of liquid biopsies (serum, plasma, PBMCs) on day -21 to -1, 7, 14, 28, 42, before surgery and at the EOSV with therapy response and clinical outcome.
Correlate immune infiltrate of resected specimen with therapy response</td></tr>
<tr><td>DESIGN / PHASE</td><td colspan="6">Prospective randomized, open-label, multicenter, phase II clinical trial</td></tr>
<tr><td>STUDY PLANNED DURATION</td><td>First patient
First visit</td><td>1Q
2020</td><td>Last patient
First visit</td><td>2Q
2022</td><td>Last patient
Last visit</td><td>4Q
2022</td></tr>
<tr><td>CENTER(S) / COUNTRY(IES)</td><td colspan="6">Vienna General Hospital, Medical University of Vienna, Vienna, Austria
State Hospital Wiener Neustadt, Lower Austria, Austria
Hospital of St. John of God, Vienna, Austria
Hospital North - Clinic Floridsdorf, Vienna, Austria
Congregational Hospital Linz - Sisters of Mercy, Linz, Austria</td></tr>
<tr><td>PATIENTS / GROUPS</td><td colspan="6">80 patients, 2 groups (50 treatment arm, 30 control arm)
Randomization ratio 30:50</td></tr>
<tr><td>INCLUSION CRITERIA</td><td colspan="6">18 years of age and older
All sexes
Histologically confirmed carcinoma of the rectum
Suitable for local therapy with curative intent
Medical need for a standard neoadjuvant CRT
Suitable to withstand a course of standard neoadjuvant CRT
Written informed consent form (ICF) for participation in the study</td></tr>
</table>

	• *Eastern Cooperative Oncology Group (ECOG) performance status of 0 or 1*
EXCLUSION CRITERIA	• *Metastatic disease that is considered incurable by local therapies*<br>• *Previous surgery of the tumor other than biopsy*<br>• *Pregnancy, breastfeeding or expectancy to conceive*<br>• *Disagreement of participants with reproductive potential to use contraception throughout the study period and for up to 180 days after the last dose of study therapy*<br>• *Prior therapy with anti-CTLA-4, anti-PD-1, anti-PD-L1, anti-PD-L2 or any other agent directed against co-inhibitory T cell receptors or has previously participated in clinical studies with immunotherapy*<br>• *Any contraindication according to the official medical information of Ipilimumab or Nivolumab*<br>• *Live vaccine within 30 days prior to the first dose of study therapy*<br>• *Hepatitis B or C*<br>• *Human immunodeficiency virus (HIV)*<br>• *Immunodeficiency*<br>• *Allogeneic tissue or solid organ transplantation*<br>• *Autoimmune disease that has required systemic therapy in the past 2 years with modifying agents, steroids or immunosuppressive drugs*<br>• *Systemic steroids or any other form of immunosuppressive therapy within 7 days prior to the first dose of study treatment*<br>• *Active non-infectious pneumonitis*<br>• *Active infection requiring systemic therapy*<br>• *Treatment with botanical preparations (i.e. herbal supplements or traditional Chinese medicines) intended for general health support or to treat the disease under study within 2 weeks prior to randomization/treatment*<br>• *Participants with serious or uncontrolled medical disorders*<br>• *Uncontrolled or significant cardiovascular disease (myocardial infarction, uncontrolled angina, any history of clinically significant arrhythmias, QTc prolongation in males > 450 ms and > 470 ms in females, participants with history of myocarditis)*<br>• *Allergies and adverse drug reaction (history of allergy or hypersensitivity to study drug components, contraindications to any of the study drugs of the chemotherapy regimen)*<br>• *Other exclusion criteria: Prisoners or participants who are involuntarily incarcerated, participants who are compulsorily detained for treatment of either a psychiatric or physical (i.e. infectious disease) illness*<br>• *White blood cells < 2000/µL (SI: < 2.00 × 109/L)*<br>• *Neutrophils < 1500/µL (SI: < 1.50 × 109/L)*<br>• *Platelets < 100 × 103/µL (SI: < 100 × 109/L) (transfusions not permitted within 72 h prior to qualifying laboratory value)*<br>• *Hemoglobin < 9.0 g/dl (SI: < 90 g/L) (transfusions not permitted within 72 h prior to qualifying laboratory value)*<br>• *Serum creatinine > 1.5 × upper limit of normal (ULN) or calculated creatinine clearance < 50 ml/min (using the Cockcroft-Gault formula)*

	• *AST/ALT: > 3.0 × ULN*<br>• *Total bilirubin > 1.5 × ULN (except participants with Gilbert Syndrome who must have a total bilirubin level of < 3.0 × ULN)*<br>• *Troponin T (TnT) or I (TnI) > 2 × institutional ULN. TnT or TnI levels between > 1 to 2 × ULN will be permitted to participate in the study if a repeat assessment remains 2 × ULN and participant undergoes a cardiac evaluation. When repeat levels within 24 h are not available, a repeat test should be conducted as soon as possible.*
STUDY PERIODS	• *Screening phase*<br>• *Treatment phase*<br>• *Follow-up phase*
INVESTIGATIONAL DRUG	**MED 1: Ipilimumab (Yervoy®)**<br>*dose: 1 mg/kg IV on day 7 (single cycle) during standard neoadjuvant CRT (50 Gy in 25 fractions with capecitabine at 1650 mg/m²/d over 25 working days)*<br><br>**MED 2: Nivolumab (Opdivo®)**<br>*dose: 3 mg/kg IV start on day 14 during standard neoadjuvant CRT (50 Gy in 25 fractions with capecitabine at 1650 mg/m²/d over 25 working days) given* every 2 weeks *until day 42 (3 cycles in total).*
COMPARATIVE DRUG / CONTROL CONDITION	*N.A.*
CONCOMITANT MEDICATION	**Allowed**<br>*N.A.*<br>**Not allowed**<br>*N.A.*
EFFICACY ENDPOINTS	*See primary and secondary objectives.*
TOLERABILITY / SAFETY ENDPOINTS	*Surgical complications will be assessed and graded according to the latest "Clavien-Dindo Classification of surgical complications".*<br>*The latest National Cancer Institute (NCI) CTCAE will be used to assess any adverse effects of the drugs (Ipilimumab, Nivolumab) used in the presents study.*
PHARMACOKINETIC / PHARMACODYNAMIC ENDPOINTS	*N.A.*
QUALITY OF LIFE / PHARMACOECONOMIC ENDPOINTS	*Quality of life will be assessed by the latest version of the European Organization for Research and Treatment of Cancer (EORCT) Quality of Life Group (QLG) questionnaires C30 and CR29.*
STATISTICAL METHODOLOGY	**Primary Endpoint**<br>*Safety, tolerability and feasibility of standard neoadjuvant CRT with sequential ipilimumab and nivolumab following surgical resection.*

Safety and tolerability will be represented by published values of known complications of CRT following surgery (Table 1).

Table 1. Incidence of know complications for neoadjuvant CRT following surgical resection.

Complications (n=128)	***Percentage (%)***	***Citation***
Any postoperative complication	*39*	*[1]*
Any surgical complication	*23*	*[1]*
Reoperation	*7*	*[1]*

Null and alternative hypotheses

H_0: Addition of sequential ipilimumab and nivolumab to standard neoadjuvant CRT is not safe and tolerable and/or increase surgical complication rates.

H_1: Addition of sequential ipilimumab and nivolumab to standard neoadjuvant CRT is safe and tolerable and does not increase surgical complication rates.

Sample size calculation

No formal sample size calculation was performed for this safety, tolerability and feasibility phase 2 study.

The 30:50 randomization ratio is a trade-off between having as much as possible patients in the treatment arm and enough patients in the control arm (standard of care treatment complication rates are well known).

The sample size of 80 is defined by the expected patient numbers available in the planned and financed duration time of the study (based on incidence, inclusion criteria and anticipated patient consent ratios). The primary objective (safety) can be compared to known and published values [1]. A doubling (100% increase) of any surgical complication (23% x 2 = 46%) can be excluded with 49 patients (Power=0.90; α=0.05).

Statistical methodology

Interim analyses will be performed after every 10 patients from the treatment arm: Reoperation numbers will be determined and the 95% CI compared to historically known and published ratios [1].

95% CI (Exact binomial test) for complication percentages between 5% and 40% in 2.5 steps and expected case numbers are calculated for n=30 (control group) and n=50 (treatment group) patients (Table 2).

Table 2. 95% CI for complication percentages and expected case numbers for each treatment group.

	n=30			***n=50***			
%	***n[1]***	***CI_{low}***	***CI_{up}***	***n[1]***	***CI_{low}***	***CI_{up}***	***% change[2]***

5	2	0	7	3	1	8	166.7
7.5	2	0	7	4	1	10	150.0
10	3	1	8	5	2	11	120.0
12.5	4	1	9	6	2	12	100.0
15	5	2	10	8	4	15	87.5
17.5	5	2	10	9	4	16	77.8
20	6	2	12	10	5	17	70.0
22.5	7	3	13	11	6	18	63.6
25	8	4	14	13	7	20	53.8
27.5	8	4	14	14	8	21	50.0
30	9	4	15	15	9	22	46.7
32.5	10	5	16	16	10	23	43.8
35	11	6	17	18	11	25	38.9
37.5	11	6	17	19	12	26	36.8
40	12	7	18	20	13	27	35.0

[1]Expected case numbers for each percentage of complication. [2]Percent change between CI_{up} and expected case numbers calcualted for the treatment group (n=50). i.e. 5%: 3 expected → 8 observed = (8-3)/3 = 166.7% change.

After end of the study the case numbers of each complication will be compared to the corresponding calculated CI_{low} and CI_{up} numbers for both groups and further rated. If in both groups the numbers are between these borders, H_0 is rejected and the treatment is considered as safe and tolerable. If the case numbers are above the CI_{up} border in the treatment group, H_0 will be accepted for this complication and the treatment is considered unsafe. If case numbers for a specific complication are below the corresponding CI_{low} border in the control group, a new complication percentage with confidence intervals for the treatment group will be calculated using the real case numbers in the control group and the case numbers in the treatment group reassessed. For the complication "reoperation" an assessment after every 10 patients of the treatment group will be performed and if the observed case numbers are above the calculated CI_{up} border case numbers, the study will be terminated (Table 3).

Table 3. Reoperation cutoff values for study termination.

n[1]	CI_{up}[2]
10	4
20	5
30	7
40	8
50	10

[1]Number of recruited patients in the treatment arm. [2]Corresponding calculated CI_{up} number of cases for the complication "reoperation" [1]. If the respective case number exceeds the calculated CI_{up} number, the study will be terminated.

3 LIST OF ABBREVATIONS

AE	Adverse Event
BRAF	V-raf murine sarcoma viral oncogene homolog B1
CI	Confidence Interval
CRC	colorectal cancer
CRO	Clinical Research Organization
CRT	Chemoradiotherapy
CSR	Clinical Study Report
CT	Computed tomography
CTCAE	Common Terminology Criteria of Adverse Events
CTLA-4	Cytotoxic T-lymphocyte-associated protein 4
DSUR	Development Safety Update Report
ECG	Electrocardiography
ECOG	Eastern Cooperative Oncology Group
eCRF	Electronic Case Report Form
EORCT	European Organization for Research and Treatment of Cancer
EOS	End of Study
EOSV	End of Study Visit
ESMO	European Society for Medical Oncology
EU	European Union
EudraCT	European Union Drug Regulating Authorities Clinical Trials
FDR	False discovery rate
GCP	Good Clinical Practice
Gy	Gray
HBV	Hepatitis B Virus
HIV	Human Immunodeficiency Virus
HVC	Hepatitis C Virus
ICH	International Conference on Harmonization
ICI	Immune checkpoint inhibitors
IEC	Independent Ethics Committee
IMP	Investigational Medicinal Product
imRECIST	immune-modified RECIST
IRB	Institutional Review Board
iRECIST	Immune RECIST
irRC	Immune-related response criteria
ISF	Investigator Site File
IV	Intravenous
KRAS	Kirsten rat sarcoma viral oncogene homolog
LARC	locally advanced rectal cancer
LLT	Lower level terms
MRI	Magnetic resonance imaging

MSI	Microsatellite instable
MSS	Microsatellite stable
N.A.	Not Applicable
NCI	National Cancer Institute
PBMCs	Peripheral blood mononuclear cells
PD-1	Programmed cell death protein 1
PD-L1	Programmed death-ligand 1
PD-L2	Programmed death-ligand 2
PET	Positron-emission tomography
PIK3CA	Phosphatidylinositol-4,5-bisphosphate 3-kinase catalytic subunit alpha
PO	Per os
PTEN	Phosphatase and tensin homolog
QLG	Quality of Life Group
RECIST 1.1	Response evaluation criteria in solid tumors 1.1
RFS	Recurrent-free survival
SAE	Serious Adverse Event
SAP	Statistical analysis plan
SAR	Serious Adverse Reaction
SOP	Standard Operating Procedure
SUSAR	Suspected Unexpected Serious Adverse Reaction
TIME	Tumor immune microenvironment
TMB	Tumor mutational burden
TP53	Tumor protein p53
Treg	Regulatory T cells
TRG	Tumor Regression Grade

4 TABLE OF CONTENTS

1 SPONSOR, INVESTIGATOR, MONITOR AND SIGNATURES 3
2 PROTOCOL SYNOPSIS 6
3 LIST OF ABBREVATIONS 11
4 TABLE OF CONTENTS 13
5 BACKGROUND INFORMATION 17
5.1 Background 17
5.2 Study rationale 17
6 STUDY OBJECTIVES 18
6.1 Primary objective 18
6.2 Secondary objectives 18
7 STUDY DESIGN 19
7.1 Study population 19
7.1.1 Subject population 19
7.1.2 Inclusion criteria 19
7.1.3 Exclusion criteria 19
7.1.4 Females of childbearing potential 20
7.1.5 Study duration 21
7.1.6 Withdrawal and replacement of subjects 21
7.1.7 Premature termination of the study 21
8 METHODOLOGY 22
8.1 Application of standard neoadjuvant CRT 22
8.2 Study medications 22
8.2.1 Investigational medicinal products (IMPs) 22
8.2.2 Standard of care (SOC) medication 23
8.2.3 Dosage and administration 23
8.2.4 Study-drug up- and down titration 23
8.2.5 Study drug interruption or discontinuation 24
8.2.6 Study drug premature permanent discontinuation 24
8.2.7 Study-drug delivery & drug storage conditions 24
8.2.8 Study drug packaging and labeling 24
8.2.9 IMP administration & handling 24
8.2.10 Drug accountability 24
8.2.11 Procedures to assess subject's compliance 24
8.2.12 Concomitant medication 25
8.3 Surgery 25
8.4 Randomization and stratification 25
8.5 Blinding 25
8.5.1 Emergency procedure for unblinding 25
8.5.2 Unblinding at the end of the study 25

8.6 Benefit and risk assessment 26
8.7 Study procedures 26
8.7.1 General rules for trial procedures 26
8.7.2 Screening investigation 26
8.7.3 Assessment of safety and tolerability 26
8.7.4 Laboratory tests 26
8.7.5 Tumor biopsies 28
8.7.6 Liquid biopsies 28
8.7.7 Stool biobanking 28
8.7.8 End-of-study (EOS) examination 29
8.7.9 Definition of the end of the trial 29
9 SAFETY DEFINITIONS AND REPORTING REQUIREMENTS 30
9.1 Surgical complications 30
9.1.1 Definitions 30
9.1.2 Severity of surgical complications 30
9.2 Adverse events (AEs) 31
9.2.1 Summary of known and potential risks of the study drug 31
9.2.2 Definition of adverse events 31
9.3 Serious adverse events (SAEs) 31
9.3.1 Hospitalization – Prolongation of existing hospitalization 32
9.3.2 SAEs related to investigational drug 33
9.3.3 Suspected unexpected serious adverse reactions (SUSARs) 33
9.3.4 Pregnancy 33
9.4 Severity of adverse events 33
9.5 Relationship to study drug 34
9.6 Reporting procedures 34
9.6.1 Reporting procedures for SUSAR 37
9.6.2 Development safety update report 38
10 FOLLOW-UP 39
10.1 Follow-up of study participants including follow-up of adverse events 39
10.2 Treatment after end of study 39
11 STATISTICAL METHODOLOGY AND ANALYSIS 40
11.1 Analysis sets 40
11.2 Sample size considerations 40
11.3 Relevant protocol deviations 40
11.4 Statistical analysis plan (SAP) 40
11.4.1 Missing, unused and spurious data 40
11.4.2 Primary endpoint analysis 41
11.4.3 Secondary endpoint analysis 41
11.4.4 Safety and tolerability endpoints 41
11.4.5 Baseline parameters and concomitant medications 41

11.4.6 Interim analysis 41
11.4.7 Software program 42
12 DOCUMENTATION AND DATA MANAGEMENT 43
12.1 Documentation of study results 43
12.1.1 Electronic case report form (eCRF) 43
12.1.2 Data collection 43
12.2 Safekeeping 43
12.3 Quality control and quality assurance 44
12.3.1 Periodic Monitoring 44
12.3.2 Audit and inspections 44
12.4 Reporting and publication 44
12.4.1 Publication of study results 44
13 ETHICAL AND LEGAL ASPECTS 45
13.1 Informed consent of subjects 45
13.2 Acknowledgement / approval of the study 45
13.2.1 Changes in the conduct of the study 45
13.3 Insurance 46
13.4 Confidentiality 46
13.5 Ethics and good clinical practice (GCP) 46
14 REFERENCES 47

Table 4. Visit and assessment schedule.

PERIODS	Name	SCREENING	TREATMENT											FOLLOW-UP
	Duration	3 weeks	17 weeks											3 weeks
VISITS	Number	1	2	3	4	5	6	7	8	9*	10*	11	12	13
	Name	Screening	Randomization	Control 1	Control 2	Control 3	Control 4	Control 5	Control 6	Control 7	Control 8	Control 9	Control 10	EOS
	Time	Day -21 to -1	Day 0	Week 1	Week 2	Week 3	Week 4	Week 5	Week 6	Week 9	Week 12	Week 15	Week 15-17	Week 20
Informed Consent		X												
Inclusion / Exclusion Criteria		X												
Medical History		X												
Physical Examination		X												
Body weight and height		X												
Vital Signs (BP, PR)		X												
12-lead ECG		X												
Pregnancy Test (monthly)		X						X						
CRT				X	X	X	X	X						
Ipilimumab				X										
Nivolumab					X		X		X					
Routine Laboratory Test		X		X	X	X	X	X	X	X	X	X	X	X
Liquid biopsy		X		X	X		X		X				X	X
Tumor biopsy		X			X		X							
Stool biobanking		X			X		X						X	
Chest-Abdomen CT scan		X												
Pelvis MRI		X										X		
Surgery													X	
Adverse Events			X	X	X	X	X	X	X	X	X	X	X	X
EORCT QLG					X		X		X			X		

*only for treatment arm (SOC+IMP)

5 BACKGROUND INFORMATION

5.1 Background

Rectal cancer accounts for approximately 125,000 cases per year in Europe [2]. Due to the narrowness of the small pelvis R0 resection remains a challenge in locally advanced rectal cancer (LARC). Neoadjuvant chemoradiotherapy (CRT) has the potential to downsize the tumor volume to increase R0 resection rates. In this line, the current European Society for Medical Oncology (ESMO) guidelines recommend CRT (45–50 Gy in 25–28 fractions for a period of 5 weeks with Capecitabine at 1650 mg/m²/d) for intermediate (cT3a/b very low, levators clear, MRF clear or cT3a/b in mid- or high rectum, cN1-2, no EMVI) and LARC (>cT3b, and EMVI+) [3].

Immune checkpoint inhibitors (ICI), such as ipilimumab (anti-cytotoxic T-lymphocyte-associated protein 4; CTLA-4) or nivolumab (anti-programmed cell death protein 1; PD-1), has proven to be an effective strategy in solid cancers, as it restores the ability of the immune system to kill cancer cells and thereby improving the rate of cure [4], [5]. However, microsatellite stable (MSS) colorectal cancer (CRC) appears to be refractory to this type of therapy, despite that the T cell-based Immunoscore® was shown to be the most relevant prognostic parameter in primary CRC [6], [7]. The fact that a small fraction (10-15%) of microsatellite instable (MSI) CRC patients respond well to ICI [6], [8], [9], indicates that CRC would be responsive to ICI in case the tumor immune microenvironment (TIME) would receive an appropriate stimuli [10]. Most recently we have demonstrated that short-course preoperative radiotherapy (SCPRT) can induce a pro-inflammatory phenotype of tumor-infiltrating macrophages in human rectal cancer patients, as well as in *ex vivo* CRC cell cultures (unpublished data). Thus, radiotherapy might induce the necessary immune activation in rectal cancer to provide an effective TIME which is necessary for ICI [11], [12].

5.2 Study rationale

The concept of combining radiotherapy with ICI is currently investigated in a variety of solid cancers including lung cancer, melanoma and head and neck cancer, as preclinical data has shown some promising synergistic effects [13]–[15]. First clinical results of small patient series support this hypothesis [16]. The safety aspect of this combination has already been documented for liver metastases of melanoma without grade 4 toxicities. Furthermore, ICI has also been combined with chemotherapy, which showed limited toxicity [17]. Yet. little data is known for the combination of CRT with sequential ICI.

Dual checkpoint therapy has been investigated in humans an appears to have additive or synergistic effects [18]–[20]. Usually, dual ICI is given at the same time. However preclinical and experimental data indicate that anti-CTLA-4 deplete regulatory T (Treg) cells and might have a priming effect for cytotoxic T cells following anti-PD-1 treatment to counterattack T cell exhaustion. Thus, the sequential blockade of the CTLA-4 and PD-1 might have and additional advantage to simultaneously inhibition.

Most recent it was shown that immunomodulating therapy applied before surgical resection are highly beneficial, which increase the recurrent-free survival (RFS) [21]–[25]. This finding suggests that systemic immune stimulation appear to purge occult micrometastases. Thus, the highest biological effects of immunotherapy might be in the early phase to tumor treatment, which focuses on the enhancement of cure rates. This provides the rational to combine ICI with currently applied standard neoadjuvant therapies in solid tumors.

6 STUDY OBJECTIVES

6.1 Primary objective

- Safety, tolerability and feasibility of sequential neoadjuvant ipilimumab and nivolumab in combination with standard neoadjuvant CRT, following surgical resection, assessed by the latest "Clavien-Dindo Classification of surgical complications" [26] (see section **9.1.2**) and Common Terminology Criteria of Adverse Events (CTCAE) [27] (see section **9.4**).

6.2 Secondary objectives

- Radiographic response determined by magnetic resonance imaging-assessed tumor regression grade (mrTRG) [28]:
  - mrTRG 1(no evidence of ever treated tumor)
  - mrTRG 2 (good response: dense fibrosis no obvious residual tumor)
  - mrTRG 3 (moderate response: >50% fibrosis or mucin & visible intermediate signal)
  - mrTRG 4 (slight response: little areas of fibrosis or mucin, but mostly tumor)
  - mrTRG 5 (no response)
- Pathological therapy response determined by tumor regression grade (TRG) [29]–[32] and the latest American Joint Committee on Cancer/International Union Against Cancer-Tumor Node Metastasis (AJCC/UICC-TNM) tumor stage [33].
  TRG according to Dworak *et al.* [32]:
  - TRG 0 (no regression)
  - TRG 1 (predominantly tumor with significant fibrosis and/or vasculopathy)
  - TRG 2 (predominantly fibrosis with scattered tumor cells)
  - TRG 3 (only scattered tumor cells in the space of fibrosis with/without acellular mucin)
  - TRG 4 (no vital tumor cells detectable)
- Correlate genomic information (*i.e.* MSI, *KRAS, BRAF, TP53, PTEN, PIK3CA*, TMB, *etc.*) with pathological and radiographic therapy response.
- Correlate genomic, transcriptomic, epigenomic and/or proteomic pattern of liquid biopsies (serum, plasma, PBMCs) taken on day -21 to -1, 7, 14, 28, 42, before surgery and at the EOSV with therapy response and clinical outcome.
- Correlate genomic, transcriptomic, epigenomic and/or proteomic pattern of tumor biopsies taken on day -21 to -1, 14 and 28 with therapy response and clinical outcome.
- Correlate immune infiltrate of resected specimen with therapy response

7 STUDY DESIGN

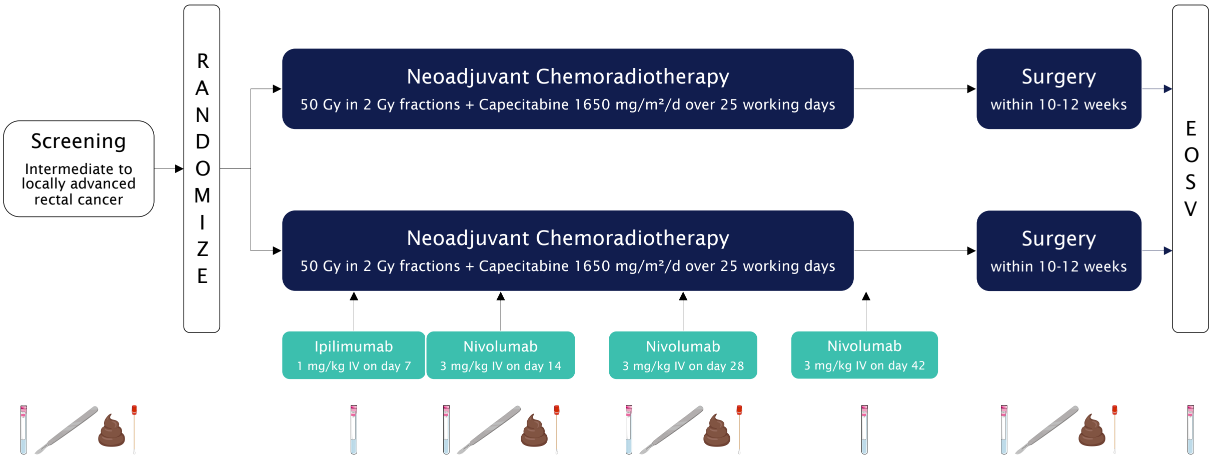

Figure 1. Study design. Prospective randomized, open-label, multicenter, phase II clinical trial. EOSV: end of study visit; IV: intravenous. Blood tube symbolizes biobanking timepoint of liquid biopsies (plasma, serum, PBMCs) and scalpel of tumor biopsies.

7.1 Study population

7.1.1 Subject population

Patients with none-metastatic rectal cancer, who need a standard neoadjuvant CRT will be enrolled in the study.

7.1.2 Inclusion criteria

- 18 years of age and older
- All sexes
- Histologically confirmed carcinoma of the rectum
- Suitable for local therapy with curative intent
- Medical need for a standard neoadjuvant CRT
- Suitable to withstand a course of standard neoadjuvant CRT
- Written informed consent form (ICF) for participation in the study
- Eastern Cooperative Oncology Group (ECOG) performance status of 0 or 1

7.1.3 Exclusion criteria

- Metastatic disease that is considered incurable by local therapies
- Previous surgery of the tumor other than biopsy
- Pregnancy, breastfeeding or expectancy to conceive
- Disagreement of participants with reproductive potential to use contraception throughout the study period and for up to 180 days after the last dose of study therapy

- Prior therapy with anti-CTLA-4, anti-PD-1, anti-PD-L1, anti-PD-L2 or any other agent directed against co-inhibitory T cell receptors or has previously participated in clinical studies with immunotherapy
- Any contraindication according to the official medical information of Ipilimumab or Nivolumab
- Live vaccine within 30 days prior to the first dose of study therapy
- Hepatitis B or C
- Human immunodeficiency virus (HIV)
- Immunodeficiency
- Allogeneic tissue or solid organ transplantation
- Autoimmune disease that has required systemic therapy in the past 2 years with modifying agents, steroids or immunosuppressive drugs
- Systemic steroids or any other form of immunosuppressive therapy within 7 days prior to the first dose of study treatment
- Active non-infectious pneumonitis
- Active infection requiring systemic therapy
- Treatment with botanical preparations (*i.e.* herbal supplements or traditional Chinese medicines) intended for general health support or to treat the disease under study within 2 weeks prior to randomization/treatment
- Participants with serious or uncontrolled medical disorders
- Uncontrolled or significant cardiovascular disease (myocardial infarction, uncontrolled angina, any history of clinically significant arrhythmias, QTc prolongation in males >450 ms and >470 ms in females, participants with history of myocarditis)
- Allergies and adverse drug reaction (history of allergy or hypersensitivity to study drug components, contraindications to any of the study drugs of the chemotherapy regimen)
- Other exclusion criteria: Prisoners or participants who are involuntarily incarcerated, participants who are compulsorily detained for treatment of either a psychiatric or physical (*i.e.* infectious disease) illness
- White blood cells < 2000/µL (SI: < 2.00×10^9/L)
- Neutrophils < 1500/µL (SI: < 1.50×10^9/L)
- Platelets < 100 × 103/µL (SI: < 100×10^9/L) (transfusions not permitted within 72 h prior to qualifying laboratory value)
- Hemoglobin < 9.0 g/dl (SI: < 90 g/L) (transfusions not permitted within 72 h prior to qualifying laboratory value)
- Serum creatinine > 1.5 × upper limit of normal (ULN) or calculated creatinine clearance < 50 ml/min (using the Cockcroft-Gault formula)
- Aspartate transaminase (AST)/Alanine transaminase (ALT): > 3.0 × ULN
- Total bilirubin > 1.5 × ULN (except participants with Gilbert Syndrome who must have a total bilirubin level of < 3.0 × ULN)
- Troponin T (TnT) or I (TnI) > 2 × institutional ULN. TnT or TnI levels between > 1 to 2 × ULN will be permitted to participate in the study if a repeat assessment remains ≤ 2 × ULN and participant undergoes a cardiac evaluation. When repeat levels within 24 h are not available, a repeat test should be conducted as soon as possible.

7.1.4 Females of childbearing potential

Females of childbearing potential must use contraception throughout the study period and for up to 180 days after the last dose of study therapy. Additionally, a pregnancy test (beta-human chorionic gonadotropin; beta-hCG) will be done monthly.

7.1.5 Study duration

The study duration for the individual participant will be approximately 20 weeks: 5 weeks of standard neoadjuvant CRT, following surgery 10 to 12 weeks after, including routine clinical and diagnostic work up of about 3 weeks.

7.1.6 Withdrawal and replacement of subjects

Criteria for withdrawal

Subjects may prematurely discontinue from the study at any time. Premature discontinuation from the study means that the subject did not undergo an end of study (EOS) examination as planned per protocol.

Subjects must be withdrawn under the following circumstances:

- at their own request
- if the Investigator feels it would not be in the best interest of the subject to continue
- if the subject violates conditions laid out in the consent form / information sheet or disregards instructions by the study personal
- in case of unacceptable toxicity
- in case of pregnancy of the subject
- if the entire study is terminated for medical or ethical reasons

In all cases, the reason why subjects are withdrawn must be recorded in detail in the eCRF and in the subject's medical records. Should the study be discontinued prematurely, all study materials (completed, partially completed and empty eCRFs) will be retained.

Follow-up of patients withdrawn from the study

In case of premature discontinuation after study drug intake, the investigations scheduled for the EOS visit will be performed 28 days after study drug discontinuation. The subjects will be advised that participation in these investigations is voluntary. Furthermore, they may request that from the time point of withdrawal no more data will be recorded and that all biological samples collected in the course of the study will be destroyed.

Replacement policy

Patients who discontinue the clinical trial before receiving the first cycle of treatment will be replaced. All other drop-outs will be included in the sample size.

7.1.7 Premature termination of the study

The sponsor has the right to close this study at any time. The IEC and the competent regulatory authority must be informed within 15 days of early termination.

The trial or single dose steps will be terminated prematurely in the following cases:

- If adverse events occur which are so serious that the risk-benefit ratio is not acceptable.
- If the number of dropouts is so high that proper completion of the trial cannot realistically be expected.

8 METHODOLOGY

8.1 Application of standard neoadjuvant CRT

Standard neoadjuvant CRT will be given according to the latest ESMO guidelines [3]. Briefly, a total of 50 Gy will be applied in 2 Gy fractions with Capecitabine 1650 mg/m²/d over 25 working days (Monday to Friday). Radiation will be delivered by 3D conformal techniques, such as intensity-modulated radiotherapy (IMRT) or volumetric-modulated arc therapy (VMAT).

8.2 Study medications

8.2.1 Investigational medicinal products (IMPs)

Active agent and characteristics: Ipilimumab

Trade name of the agent: Yervoy®

Manufacturer: Bristol-Myers Squibb

Drug supply: Ipilimumab is supplied as a Clear to slightly opalescent, colorless to pale yellow liquid and has a pH of 7.0 and an osmolarity of 260-300 mOsm/kg. Each ml of concentrate contains 5 mg ipilimumab. Excipients are tris hydrochloride, sodium chloride, mannitol, pentetic acid, polysorbate 80, sodium hydroxide, hydrochloric acid and water for injections.

Storage Instructions: Store in a refrigerator (2°C to 8°C). Do not freeze. Store in the original package in order to protect from light. From a microbiological point of view, once opened, the medicinal product should be infused or diluted and infused immediately. The chemical and physical in-use stability of the undiluted or diluted concentrate (between 1 and 4 mg/ml) has been demonstrated for 24 h at 25°C and 2°C to 8°C. If not used immediately, the infusion solution (undiluted or diluted) may be stored for up to 24 hours in a refrigerator (2°C to 8°C) or at room temperature (20°C to 25°C).

Route of administration: IV over 30 minutes

Further specifications can be found in the investigator's brochure (IB) for Ipilimumab (section 5.6).

Active agent and characteristics: Nivolumab

Trade name of the agent: Opdivo®

Manufacturer: Bristol-Myers Squibb

Drug supply: Nivolumab is supplied as a clear to opalescent, colorless to pale yellow liquid. The solution has a pH of approximately 6.0 and an osmolality of approximately 340 mOsm/kg. Each ml of concentrate contains 10 mg of nivolumab. Excipients are sodium citrate dihydrate, sodium chloride, mannitol, pentetic acid, polysorbate 80, sodium hydroxide, hydrochloric acid and water for injections.

Storage Instructions: Store in a refrigerator (2°C to 8°C). Do not freeze. Store in the original package in order to protect from light. From a microbiological point of view, the product should be used immediately. If not used immediately, chemical and physical in-use stability has been demonstrated for 24 h at 2°C to 8°C protected from light and a maximum of 8 h at 20°C to 25°C and room light (this 8-hour period of the total 24 h should be inclusive of the product administration period).

Route of administration: IV over 30 minutes

Further specifications can be found in the IB for nivolumab (section 5.6).

8.2.2 Standard of care (SOC) medication

Active agent and characteristics: Capecitabine

Trade name of the agent: generic

Manufacturer: generic

Drug supply: generic specific

Storage Instructions: generic specific

Route of administration: PO

Further specifications can be found in the generic specific SmPC.

8.2.3 Dosage and administration

Initial dose: Ipilimumab 1 mg/kg on day 7 during standard neoadjuvant CRT

Maintenance dose: N.A.

Route of administration: IV over 30 minutes

Duration: single dose

Initial dose: Nivolumab 3 mg/kg on day 14 during standard neoadjuvant CRT

Maintenance dose: 3 mg/kg

Route of administration: IV over 30 minutes

Duration: given every 2 weeks until day 42 (3 cycles in total)

Initial dose: Capecitabine 1650 mg/m^2/d given as two equal doses per day concurrent with standard neoadjuvant CRT

Maintenance dose: 1650 mg/m^2/d given as two equal doses per day

Route of administration: PO

Duration: given concurrent with standard neoadjuvant CRT over 25 working days

8.2.4 Study-drug up- and down titration

There will be no up and down titration or dose modifications of the investigational drugs.

8.2.5 Study drug interruption or discontinuation

The Investigator must temporarily interrupt or permanently discontinue the study drug according to the specifications in the drug specific SmPC.

The reason for study drug interruption or premature permanent discontinuation must be documented in the eCRF.

8.2.6 Study drug premature permanent discontinuation

Study drug premature permanent discontinuation due to an adverse event

If the reason for premature permanent discontinuation of study treatment is due to a specification in the drug specific SmPC, the patient should have a “Premature End of Study (EOS)” visit with all the assessments performed before the study drug discontinuation, whenever possible.

8.2.7 Study-drug delivery & drug storage conditions

The investigational drugs will be provided by Bristol-Myers Squibb and stored at the hospital pharmacies of the Vienna General Hospital (AKH) according to the storage conditions specified in the SmPC.

8.2.8 Study drug packaging and labeling

Investigational drugs will be provided and packed Bristol-Myers Squibb. Investigational drugs will be labeled by the institutional pharmacies of the trial centers.

8.2.9 IMP administration & handling

The investigational drugs will be administered and handled according to the specifications in the SmPC.

8.2.10 Drug accountability

The investigational drugs will be requested from the hospital pharmacies of the Vienna General Hospital (AKH) for each study participant separately and administered promptly. Drug dispensing will be documented by site staff.

8.2.11 Procedures to assess subject’s compliance

Not applicable due to IV administration of the investigational drugs by medical personnel.

8.2.12 Concomitant medication

The use of systemic corticosteroids and other immunosuppressants at baseline, before starting nivolumab or ipilimumab, should be avoided because of their potential interference with the pharmacodynamic activity. However, systemic corticosteroids and other immunosuppressants can be used after starting nivolumab to treat immune-related adverse reactions. The preliminary results show that systemic immunosuppression after starting nivolumab treatment does not appear to preclude the response on nivolumab or ipilimumab.

The use of anticoagulants is known to increase the risk of gastrointestinal hemorrhage. Since gastrointestinal hemorrhage is an adverse reaction with ipilimumab, patients who require concomitant anticoagulant therapy should be monitored closely.

Any concomitant medication should be documented in the patient's records. The use of concomitant medications should follow standard medical oncology practice.

8.3 Surgery

Patients will be operated about 10-12 weeks post CRT. Surgical options included standard total mesorectal excision (TME) or extended TME (*i.e.* TME with adjacent visceral resection) with or without sphincter preservation (abdominoperineal excision; APE) according to a standardized technique [34][3]. Patients will receive either a minimal invasive (laparoscopic, laparoscopic-assisted or robotic) or an open approach, depending on the perioperative circumstances (*i.e.* body constitution and cardiopulmonary fitness of the patient) and the surgeon's choice and expertise.

8.4 Randomization and stratification

On day 0 patients will be randomized to 1 of the 2 study groups using a "permuted block randomization" with a block size of 8 (https://www.meduniwien.ac.at/randomizer).

8.5 Blinding

Not applicable due to the open-label study design.

8.5.1 Emergency procedure for unblinding

Not applicable due to the open-label study design.

8.5.2 Unblinding at the end of the study

Not applicable due to the open-label study design.

8.6 Benefit and risk assessment

As this study is primary designed for the assessment of safety, tolerability and feasibility of a single dose of neoadjuvant ipilimumab and following 3 cycles of nivolumab in combination with standard neoadjuvant CRT in rectal cancer, there is no explicit benefit for the individual participant. Other interventions, such as blood drawing, tumor biopsies, computed tomography (CT), magnetic resonance imaging (MRI), positron-emission tomography (PET), neoadjuvant CRT, following surgical resection are standard care of treatment for rectal cancer patients. However, it is expected that patients within the treatment arm are going to have a major benefit in terms of pathological response and consequently clinical outcome.

8.7 Study procedures

8.7.1 General rules for trial procedures

- All study measures like blood sampling and measurements (vital parameters, ECG, *etc.*) have to be documented with date (dd.mm.yyyy).
- In case several study procedures are scheduled at the same time point, there is no specific sequence that should be followed.
- The dates of all procedures should be according to the protocol. The time margins mentioned in the study flow chart are admissible. If for any reason, a study procedure is not performed within scheduled margins a protocol deviation should be noted, and the procedure should be performed as soon as possible or as adequate.
- If it is necessary for organizational reasons, it is admissible to perform procedures which are scheduled for one visit at two different time points. Allowed time margins should thereby not be exceeded.

8.7.2 Screening investigation

Screening investigations will be done according to standard routine surgical practice for patients with rectal cancer who need a neoadjuvant CRT [3].

8.7.3 Assessment of safety and tolerability

Safety, tolerability and feasibility assessed by the latest "Clavien-Dindo Classification of surgical complications" [26] (see section **9.1.2**) and CTCAE [27] (see section **9.4**).

8.7.4 Laboratory tests

Laboratory test and procedures will be done according to standard clinical practice for patients with rectal cancer who receive a neoadjuvant CRT following surgical resection [3].

- Blood count:

    - Erythrocytes
    - Hemoglobin
    - Thrombocytes
    - Leucocytes
        - Neutrophils
        - Lymphocytes
        - Monocytes
        - Eosinophils
        - Basophils
- Coagulation:
    - Thromboplastin time (PTZ)
    - International normalized ratio (INR)
    - Partial thromboplastin time (aPPT)
- Kidney:
    - Creatinine
    - Blood urea nitrogen (BUN)
- Liver/Pancreas
    - Aspartate aminotransferase (ASAT) / Glutamic oxaloacetic transaminase (GOT)
    - Alanine aminotransferase (ALAT) / Glutamate-pyruvate transaminase (GPT)
    - Gamma-glutamyltransferase (Gamma-GT)
    - Alkaline phosphatase (AP)
    - Bilirubin
    - Pancreatic amylase
    - Lipase
    - Lactate dehydrogenase (LDH)
- Protein diagnostic
    - Albumin
- Inflammation
    - C-reactive protein (CRP)
- Glucose metabolism
    - Glucose
    - Glycated hemoglobin (HbA1c)
- Thyroid diagnostic
    - Thyroid-stimulating hormone (TSH)
    - Free thyroxine (fT4)
    - Free triiodothyronine (fT3)
- Cardiac diagnostic
    - Troponin T (TnT)
    - Creatine kinase (CK)
    - Creatine kinase muscle-brain (CK-MB)
    - NT pro brain natriuretic peptide (proBNP)
- Hormones
    - Beta-human chorionic gonadotropin (beta-hCG)
- Tumor marker
    - Carcinoembryonic antigen (CEA)
    - Carbohydrate antigen 19-9 (CA 19-9)

8.7.5 Tumor biopsies

Additional biobanking will be done from pretherapeutic biopsies (day -21 to -1), which are collected anyhow for routing clinical diagnostic, as well as from tumor resected specimen (between week 15 to 17). Additional study specific interventions would include tumor biopsies on day 14 and 28. Tissue biobanking material will be used for genomic, transcriptomic, epigenomic and/or proteomic investigations/correlations with therapy response and clinical outcome.

Genomic analysis of circulating tumor DNA (ctDNA) and tumor tissue DNA will include standard high throughput next generation sequencing (NGS) technologies, such as whole genome sequencing (WGS) and/or whole exome sequencing (WES).

Transcriptomic analysis will include standard high-throughput technologies, such as single-cell RNA sequencing (scRNA-seq), single-cell T-cell receptor sequencing (scTCR-seq), single-cell B-cell receptor sequencing (scBCR-seq), single nuclei RNA sequencing (snRNA-seq), spatial transcriptomics (Visium Spatial platform, 10X Genomics), cellular indexing of transcriptomes and epitopes by sequencing (CITE-seq) [35] and/or RNA expression and protein sequencing assay (REAP-seq) [36].

Epigenomic analysis will include reduced representation bisulfite sequencing (RRBS) [37] and/or chromatin immunoprecipitation DNA-sequencing (ChIP-seq) [38]–[40].

Proteomic and metabolomic will include standard high-throughput technologies, such as orbitrap mass spectrometry, matrix assisted laser desorption/ionization (MALDI), electrospray ionization (ESI), liquid chromatography–mass spectrometry (LC–MS), gas chromatography–mass spectrometry (GC-MS) and/or cytometry by time of flight (CyTOF) [41]. Protein analysis of single cell suspensions will include standardized flow cytometry (FCM) techniques. Tissue protein analysis will include standard immunohistochemistry (IHC) and/or multiplex immunofluorescence assays (*i.e.* Akoya Biosciences). Stained tissue sections will be quantified by whole-slide tissue scanning systems (*i.e.* Vectra Polaris, PerkinElmer) and further analyzed with an image analysis software (*i.e.* HALO, Indica Labs).

All multi-omic analysis will be assessed longitudinal for intra/inter tumor heterogeneity and further correlated with therapy response and clinical outcome. Moreover, samples will be compared between the 2 different patient groups (control arm vs. treatment arm).

All analysis will be only done by or in cooperation with local and/or international non-profit/none-commercial academic centers, such as universities or research institutions. *i.e.* VIB KU Leuven Center for Cancer Biology (Diether Lambrechts), Research Center for Molecular Medicine (CeMM) of the Austrian Academy of Sciences (Christoph Bock), Joint Microbiome Facility (JMF) of the Medical University of Vienna and the University of Vienna (David Berry) and/or members of the Society for Immunotherapy of Cancer (SITC).

8.7.6 Liquid biopsies

Study specific interventions include biobanking of liquid biopsies (serum, plasma, PBMCs) on day -21 to -1, 7, 14, 28, 42, before surgery and at the EOSV. Blood samples are tried to be collected during routine blood drawing. Biobanking material (blood and tissue) will be used for genomic, transcriptomic, epigenomic and/or proteomic investigations/correlations with therapy response and clinical outcome.

Analysis of longitudinal liquid biopsies will be analyzed according to the above-mentioned tumor tissue biopsies.

8.7.7 Stool biobanking

Additional stool biobanking will be done concomitant with the tumor biopsy interventions described above in section 8.7.5 Stool biobanking material will be used for metagenomics, metatranscriptomics,

metaproteomics and/or metabolomics investigations/correlations with therapy response and clinical outcome.

Metagenomic analysis will include high-throughput technologies, such as highly multiplexed gene amplicon sequencing (*i.e.* 16S rRNA genes).

Metatranscriptomics will be done by high-throughput technologies, such as Illumina HiSeq-/NovaSeq.

Metabolomics will be analyzed by LC–MS, GC-MS and/or nuclear magnetic resonance (NMR) spectroscopy.

Analysis of longitudinal meta-omics data will be complemented with functional assays, such as bacterial cultivation of harvested stool samples/swabs and further correlated with therapy response and clinical outcome. Moreover, samples will be compared between the 2 different patient groups (control arm vs. treatment arm).

8.7.8 End-of-study (EOS) examination

After successful surgery (approximately 20 weeks after treatment period) patients undergo the EOS examination, which entails a standard patient visit according to routine surgical practice.

8.7.9 Definition of the end of the trial

The end of the trial is defined as the date of the last visit of the last patient undergoing the trial.

9 SAFETY DEFINITIONS AND REPORTING REQUIREMENTS

9.1 Surgical complications

9.1.1 Definitions

Surgical complications are defined as any deviation from the normal postoperative course. This definition also considers asymptomatic complications such as arrhythmia and atelectasis.

A sequela is an “after-effect” of surgery that is inherent to the procedure (*i.e.* inability to walk after an amputation of the leg).

Finally, surgery may be well executed without any complications but still fail. If the original purpose of surgery has not been achieved, this is not a complication but a “failure to cure” (*i.e.* residual tumor after surgery). Sequelae and failure to cure should not be included in the new classification of complications.

9.1.2 Severity of surgical complications

Surgical complications will be assessed and graded according to the latest “Clavien-Dindo Classification of surgical complications” [26]. Briefly, it consists of 7 grades (I, II, IIIa, IIIb, IVa, IVb and V). Subclasses a and b can be omitted to contract the classification into 5 grades (I, II, III, IV and V), depending on the size of the population and/or the of the emphasis of a study.

Grade I

Any deviation from the normal postoperative course without the need for pharmacological treatment or surgical, endoscopic and radiological interventions. Allowed therapeutic regimens are: drugs as antiemetics, antipyretics, analgetics, diuretics and electrolytes and physiotherapy. This grade also includes wound infections opened at the bedside.

Grade II

Requiring pharmacological treatment with drugs other than such allowed for grade I complications. Blood transfusions and total parenteral nutrition are also included.

Grade III

Requiring surgical, endoscopic or radiological intervention

Grade IIIa

Intervention not under general anesthesia

Grade IIIb

Intervention under general anesthesia

Grade IV

Life-threatening complication (including CNS complications) requiring IC/ICU-management

Grade IVa

Single organ dysfunction (including dialysis)

Grade IVb

Multiple organ dysfunction

Grade V

Death of a patient

9.2 Adverse events (AEs)

9.2.1 Summary of known and potential risks of the study drug

The overall safety experience with nivolumab, as monotherapy or in combination with ipilimumab, is based on experience in approximately 12,300 subjects treated to date. Extensive details on the safety profile of nivolumab and ipilimumab, including results from other clinical studies, are available in section 5.6 of the nivolumab IB or ipilimumab IB and will not be repeated herein.

Overall, the safety profile of nivolumab in combination with ipilimumab or radiotherapy is manageable and generally consistent across completed and ongoing clinical trials. Most AEs were low-grade (Grade 1 to 2) with relatively few related high-grade (Grade 3 to 4) AEs. There was no pattern in the incidence, severity, or causality of AEs with respect to nivolumab dose level. Results to date suggest that the safety profile of nivolumab/ipilimumab combination therapy and nivolumab in combination with radiotherapy is consistent with the mechanisms of action of nivolumab and ipilimumab. The nature of the AEs is similar to that observed with either agent used as monotherapy; however, both frequency and severity of most AEs are increased with the combination.

A pattern of immune-related AEs has been defined, for which management algorithms have been developed; these are provided in nivolumab IB/SmPC or ipilimumab IB/SmPC. Most high-grade events were manageable with the use of corticosteroids or hormone replacement therapy (endocrinopathies) as instructed in these algorithms. For additional material, see the nivolumab IB/SmPC and ipilimumab IB/SmPC.

9.2.2 Definition of adverse events

An adverse event (AE) is defined as any new untoward medical occurrence or worsening of a preexisting medical condition in a clinical investigation participant administered study drug and that does not necessarily have a causal relationship with this treatment. An AE can therefore be any unfavorable and unintended sign (such as an abnormal laboratory finding), symptom, or disease temporally associated with the use of investigational product, whether or not considered related to the investigational product.

A ***non-serious adverse event*** is an AE not classified as serious.

9.3 Serious adverse events (SAEs)

A serious adverse event (SAE) is any untoward medical occurrence that at any dose:

- results in death

- is life-threatening (defined as an event in which the participant was at risk of death at the time of the event; it does not refer to an event which hypothetically might have caused death if it were more severe)
- requires inpatient hospitalization or causes prolongation of existing hospitalization (see **NOTE** below)
- results in persistent or significant disability/incapacity
- is a congenital anomaly/birth defect
- is an important medical event (defined as a medical event(s) that may not be immediately life-threatening or result in death or hospitalization but, based upon appropriate medical and scientific judgment, may jeopardize the subject or may require intervention [eg, medical, surgical] to prevent one of the other serious outcomes listed in the definition above.) Examples of such events include, but are not limited to, intensive treatment in an emergency room or at home for allergic bronchospasm; blood dyscrasias or convulsions that do not result in hospitalization.)
- Suspected transmission of an infectious agent (*i.e.* pathogenic or nonpathogenic) via the study drug is an SAE.

Although pregnancy and potential drug-induced liver injury (DILI), are not always serious by regulatory definition, however, these events must be reported within the SAEs timeline.

Any component of a study endpoint that is considered related to study therapy should be reported as an SAE (*i.e.* death is an endpoint, if death occurred due to anaphylaxis, anaphylaxis must be reported).

9.3.1 Hospitalization – Prolongation of existing hospitalization

Hospitalization is defined as an overnight stay in a hospital unit and/or emergency room. An additional overnight stay defines a prolongation of existing hospitalization.

The following is not considered an SAE and should be reported as an AE only:

- Treatment on an emergency or outsubject basis for an event not fulfilling the definition of seriousness given above and not resulting in hospitalization.

The following reasons for hospitalizations are not considered AEs, and therefore not SAEs:

- Hospitalizations for cosmetic elective surgery, social and/or convenience reasons.
- Elective treatment of a pre-existing disease or medical condition that did not worsen, *i.e.* hospitalization for chemotherapy for cancer, elective hip replacement for arthritis.
- A visit to the emergency room or other hospital department < 24 h, that does not result in admission (unless considered an important medical or life-threatening event)
- Elective surgery, planned prior to signing consent
- Admissions as per protocol for a planned medical/surgical procedure
- Routine health assessment requiring admission for baseline/trending of health status (*i.e.* routine colonoscopy)

- Medical/surgical admission other than to remedy ill health and planned prior to entry into the study. Appropriate documentation is required in these cases.
- Admission encountered for another life circumstance that carries no bearing on health status and requires no medical/surgical intervention (*i.e.* lack of housing, economic inadequacy, caregiver respite, family circumstances, administrative reason).
- Admission for administration of anticancer therapy in the absence of any other SAEs (applies to oncology protocols)

9.3.2 SAEs related to investigational drug

Such SAEs are defined as SAEs that appear to have a reasonable possibility of causal relationship.

9.3.3 Suspected unexpected serious adverse reactions (SUSARs)

SUSARs are all serious adverse reactions with **suspected** causal relationship to the study drug that is **unexpected** (not previously described in the SmPC or IB) and serious.

9.3.4 Pregnancy

If, following initiation of the investigational product, it is subsequently discovered that a study participant is pregnant or may have been pregnant at the time of investigational product exposure, including during at least 5 half-lives after product administration, the investigational product will be permanently discontinued in an appropriate manner (*i.e.* dose tapering if necessary for participant).

The investigator must immediately notify Worldwide.Safety@bms.com of this event and complete one of the following forms within 24 h of awareness of the event via either the CIOMS, MedWatch or appropriate Pregnancy Surveillance Form in accordance with SAE reporting procedures.

Protocol-required procedures for study discontinuation and follow-up must be performed on the participant.

Follow-up information regarding the course of the pregnancy, including perinatal and neonatal outcome and, where applicable, offspring information must be reported on the CIOMS, MedWatch, BMS Pregnancy Surveillance Form, **or** approved site SAE form. A BMS Pregnancy Surveillance Form may be provided upon request.

Any pregnancy that occurs in a female partner of a male study participant should be reported to BMS. Information on this pregnancy will be collected on the Pregnancy Surveillance Form. In order for Sponsor or designee to collect any pregnancy surveillance information from the female partner, the female partner must sign an informed consent form for disclosure of this information.

9.4 Severity of adverse events

The severity of clinical AEs is graded on the latest Common Terminology Criteria for Adverse Events (CTCAE) [27] and reported on specific AE pages of the eCRF.

Grade 1

Mild; Asymptomatic or mild symptoms; clinical or diagnostic observations only; intervention not indicated.

Grade 2

Moderate; Minimal, local or noninvasive intervention indicated; limiting age-appropriate instrumental Activities of Daily Living (ADL; refer to preparing meals, shopping for groceries or clothes, using the telephone, managing money, etc.

Grade 3

Severe or medically significant but not immediately life-threatening; Hospitalization or prolongation of hospitalization indicated; disabling; limiting self-care ADL (refer to bathing, dressing and undressing, feeding self, using the toilet, taking medications, and not bedridden).

Grade 4

Life-threatening consequences; Urgent intervention indicated.

Grade 5

Death related to AE.

9.5 Relationship to study drug

For all AEs, the Investigator will assess the causal relationship between the study drug and the AE using his/her clinical expertise and judgment according to the following algorithm that best fits the circumstances of the AE:

Not related

- May or may not follow a temporal sequence from administration of the study product
- Is biologically implausible and does not follow known response pattern to the suspect study drug (if response pattern is previously known).
- Can be explained by the known characteristics of the subject's clinical state or other modes of therapy administered to the subject.

Related

- Reasonable temporal relation between the AE and the intake of the study medication and
- There is no other explanation for the AE and
- Subsidence or disappearance of the AE on withdrawal of the study medication and
- Recurrence of the symptoms on restart at previous dose (only applies for re-institution of mediation).

9.6 Reporting procedures

- All Serious Adverse Events (SAEs) that occur following the subject's written consent to participate in the study through 100 days of discontinuation of dosing must be reported to BMS Worldwide

Safety, whether related or not related to study drug. If applicable, SAEs must be collected that relate to any later protocol-specified procedure (*i.e.* a follow-up biopsy).

- Following the subject's written consent to participate in the study, all SAEs, whether related or not related to study drug, are collected, including those thought to be associated with protocol-specified procedures. The investigator should report any SAE occurring after these aforementioned time periods, which is believed to be related to study drug or protocol-specified procedure.
- An SAE report should be completed for any event where doubt exists regarding its seriousness;
- If the investigator believes that an SAE is not related to study drug, but is potentially related to the conditions of the study (such as withdrawal of previous therapy or a complication of a study procedure), the relationship should be specified in the narrative section of the SAE Report Form.

An appropriate SAE form (*i.e.* ex-US = CIOMS form or USA = Medwatch form) should be used to report SAEs to BMS. If you prefer to use your own Institutional form, it must be reviewed by the BMS Protocol Manager prior to study initiation to ensure that at a minimum all of the data elements on the CIOMS form are present. The BMS Protocol number will be included on the SAE form or on the cover sheet with the SAE form transmission.

- The CIOMS form is available at: http://www.cioms.ch/index.php/cioms-form-i
- The MedWatch form is available at: MedWatch 3500 Form
- For studies with long-term follow-up periods in which safety data are being reported, include the timing of SAE collection.
- The Sponsor will reconcile the clinical database AE cases (**case level only**) transmitted to BMS Global Pharmacovigilance (Worldwide.Safety@bms.com).
    - The Investigator will request from BMS GPV&E, aepbusinessprocess@bms.com the SAE reconciliation report and include the BMS protocol number every 3 months and prior to data base lock or final data summary
    - GPV&E will send the investigator the report to verify and confirm all SAEs have been transmitted to BMS GPV&E.
    - The data elements listed on the GPV&E reconciliation report will be used for case identification purposes. If the Investigator determines a case was not transmitted to BMS GPV&E, the case should be sent immediately to BMS (Worldwide.Safety@bms.com).
- In addition to the Sponsor Investigator's responsibility to report events to their local HA, suspected serious adverse reactions (whether expected or unexpected) shall be reported by BMS to the relevant competent health authorities in all concerned countries according to local regulations (either as expedited and/or in aggregate reports).
- In accordance with local regulations, BMS will notify sponsor investigators of all reported SAEs that are suspected (related to the investigational product) and unexpected (*i.e.* not previously described in the IB). An event meeting these criteria is termed a Suspected, Unexpected Serious Adverse Reaction (SUSAR). Sponsor investigator notification of these events will be in the form of either a SUSAR Report or a Semi-Annual SUSAR Report.
    - Other important findings which may be reported by BMS as an Expedited Safety Report (ESR) include: increased frequency of a clinically significant expected SAE, an SAE considered associated with study procedures that could modify the conduct of the study, lack of efficacy that poses significant hazard to study subjects, clinically significant safety finding from a nonclinical (*i.e.* animal) study, important safety recommendations

from a study data monitoring committee, or sponsor or BMS decision to end or temporarily halt a clinical study for safety reasons.

- Upon receiving an ESR from BMS, the investigator must review and retain the ESR with the IB. Where required by local regulations or when there is a central IRB/IEC for the study, the sponsor will submit the ESR to the appropriate IRB/IEC. The investigator and IRB/IEC will determine if the informed consent requires revision. The investigator should also comply with the IRB/IEC procedures for reporting any other safety information.

SAEs, whether related or not related to study drug, and pregnancies must be reported to BMS within 24 h \ 1 Business Day of becoming aware of the event. SAEs must be recorded on either CIOMS, MedWatch, or approved site SAE form.

Pregnancies must be reported and submitted to BMS. BMS will perform due diligence follow-up using the BMS Pregnancy Form which the investigator must complete.

SAE Email Address: Worldwide.Safety@BMS.com

SAE Facsimile Number: +1 609-818-3804

If only limited information is initially available, follow-up reports are required. (Note: Follow-up SAE reports should include the same investigator term(s) initially reported.)

If an ongoing SAE changes in its intensity or relationship to study drug or if new information becomes available, a follow-up SAE report should be sent within 24 h \ 1 Business Day to BMS using the same procedure used for transmitting the initial SAE report.

All SAEs should be followed to resolution or stabilization.

The causal relationship to study drug is determined by a physician and should be used to assess all adverse events (AE). The casual relationship can be one of the following:

Related: There is a reasonable causal relationship between study drug administration and the AE.

Not related: There is not a reasonable causal relationship between study drug administration and the AE.

The term "reasonable causal relationship" means there is evidence to suggest a causal relationship.

Adverse events can be spontaneously reported or elicited during open-ended questioning, examination, or evaluation of a subject. (In order to prevent reporting bias, subjects should not be questioned regarding the specific occurrence of one or more AEs.)

Non-serious Adverse Event

- Non-serious Adverse Events (AE) are to be provided to BMS in aggregate via interim or final study reports as specified in the agreement or, if a regulatory requirement [*i.e.* IND US trial] as part of an annual reporting requirement.
- Non-serious AE information should also be collected from following the subject's written consent to participate in the study.

Non-serious Adverse Event Collection and Reporting

The collection of non-serious AE information should begin following the subject's written consent to participate in the study. All non-serious adverse events (not only those deemed to be treatment-related) should be collected continuously during the treatment period and for a minimum of 100 days following the last dose of study treatment.

Non-serious AEs should be followed to resolution or stabilization, or reported as SAEs if they become serious. Follow-up is also required for non-serious AEs that cause interruption or discontinuation of study drug and for those present at the end of study treatment as appropriate.

Laboratory Test Abnormalities

All laboratory test results captured as part of the study should be recorded following institutional procedures. Test results that constitute SAEs should be documented and reported to BMS as such.

The following laboratory abnormalities should be documented and reported appropriately:

- any laboratory test result that is clinically significant or meets the definition of an SAE
- any laboratory abnormality that required the participant to have study drug discontinued or interrupted
- any laboratory abnormality that required the subject to receive specific corrective therapy.

It is expected that wherever possible, the clinical rather than laboratory term would be used by the reporting investigator (*i.e.* anemia versus low hemoglobin value).

Other Safety Considerations

Any significant worsening noted during interim or final physical examinations, electrocardiograms, X-rays, and any other potential safety assessments, whether or not these procedures are required by the protocol, should also be recorded as a non-serious or serious AE, as appropriate, and reported accordingly.

A special section is designated to adverse events in the eCRF. The following details must thereby be entered:

- Type of adverse event
- Start (date and time)
- End (date and time)
- Severity (Grad 1-5)
- Serious (no / yes)
- Unexpected (no / yes)
- Outcome (resolved, resolving, not resolved, resolved with sequelae, unknown, fatal)
- Relation to study drug (Related / Not related)

Adverse events are to be documented in the eCRF in accordance with the above-mentioned criteria.

9.6.1 Reporting procedures for SUSAR

It must be remembered that the regulatory authorities, and the Institutional Review Board / Independent Ethics Committee (IRB / IEC) must be informed about all SUSAR. Such reports shall be made by the sponsor and should content at least the following details:

- Patient number (study code/screening number)
- Patient: age in years, sex
- Name of Investigator and investigating site
- Period of administration
- The suspected investigational medical product (IMP)

- The adverse event assessed as serious and unexpected, and for which there is a **suspected** causal relationship to the IMP
- Concomitant disease and medication
- Short description of the event:
    - Description
    - Onset and if applicable, end
    - Therapeutic intervention
    - Causal relationship
    - Seriousness criteria or reportable reason

Electronic reporting should be the expected method for reporting of SUSARs to the competent authority. In that case, the format and content as defined by the regulatory requirements should be adhered to. The latest version of MedDRA should be applied. Lower level terms (LLT) should be used.

9.6.2 Development safety update report

A Development Safety Update Report (DSUR) will be provided by the Sponsor annually.

This report will also be presented annually to the Independent Ethics (IEC) and to the competent authorities by the sponsor, as well as to BMS.

10 FOLLOW-UP

10.1 Follow-up of study participants including follow-up of adverse events

Study specific follow-up for safety, tolerability and feasibility will end after EOS. Nevertheless, patients will undergo standard oncologic follow-up, including recurrence-free survival (RFS) and overall survival (OS), according to the latest ESMO guidelines for local/locoregional rectal cancer [3].

10.2 Treatment after end of study

There will be no study related treatment after end of study.

11 STATISTICAL METHODOLOGY AND ANALYSIS

11.1 Analysis sets

Treatment set

This analysis set includes all subjects who were randomized to the treatment arm and received neoadjuvant CRT with sequential Ipilimumab and Nivolumab and did not violate the protocol in a way that might affect the evaluation of the effect of the study drugs on the primary objective.

Control set

This analysis set includes all subjects who were randomized to the control arm and received neoadjuvant CRT and did not violate the protocol in a way that might affect the evaluation of the effect of the study drugs on the primary objective.

11.2 Sample size considerations

No formal sample size calculation was performed for this safety, tolerability and feasibility phase 2 study.

Nevertheless, an informal power analysis using published data [1] reveals that an increase of 100% of surgical complications (=46%) can be excluded with 49 patients in the treatment arm (Power=0.90, α=0.05), compared to the published 23%).

11.3 Relevant protocol deviations

All protocol deviations will be listed in the study report.

11.4 Statistical analysis plan (SAP)

11.4.1 Missing, unused and spurious data

In general, every effort shall be made by site staff to avoid the occurrence of missing data. However, in case data is not recorded, this must be clearly stated in the eCRF. The statistical analysis will also report the frequency of missing values.

Patients with missing data for a specific side effect will be excluded from the evaluation of this side effect. Missing data for secondary objectives will be imputed with values estimated by the predictive mean matching method using R-package “mice” (version 2.30), if appropriate.

11.4.2 Primary endpoint analysis

Safety and tolerability will be assessed by comparing case numbers for any complication in the treatment arm (n=50) with the calculated 95% upper CI numbers of the calculated percentage of expected numbers of current standard therapy (Table 2). Case numbers in the control arm (n=30) will be used to validate expected numbers from current standard therapy (*i.e.* will be compared to the corresponding 95% CI lower and upper borders).

11.4.3 Secondary endpoint analysis

Statistical analysis of secondary objectives, especially between control and treatment arm will be performed according the nature of the outcome parameter. Nominal and dichotomous, outcomes will be compared by Chi-squared or Fisher's exact tests, continuous variables will be compared by T-tests (after $\log_2$ transformation) or by Mann-Whitney-U tests and censored survival outcomes by Cox-regression analysis. Potential confounders will be considered in corresponding statistical models. Multiple testing will be corrected for by the Holm's Step-Down method or by the false discovery rate (FDR) method after Benjamini-Hochberg (especially omics data) [42]. Adjusted p-values (q-values) below 0.05 will be considered as statistically significant.

11.4.4 Safety and tolerability endpoints

The safety analysis will be performed in all participants who have received at least 1 dose of study treatment. Descriptive statistics of safety will be presented using latest NCI CTCAE by treatment arm. Adverse events, treatment-related AEs, SAEs and treatment-related SAEs will be tabulated using worst grade per latest NCI CTCAE criteria by system organ class and preferred term. On-study lab parameters including hematology, chemistry including liver function and renal function will be summarized using worst grade per latest NCI CTCAE criteria.

11.4.5 Baseline parameters and concomitant medications

Baseline parameters, such as age, sex, tumor stage, etc. will be included as confounder into the analyses of secondary objectives, if appropriate. For safety and tolerability analyses, these parameters will not be considered.

11.4.6 Interim analysis

Interim analyses will be performed after every 10 patients from the treatment arm to assess the reoperation complication rate compared to CI of known ratios. If the 95% CI upper limit gets exceeded at any time, the study will be terminated.

Criteria for the termination of the trial

The study will be terminated if the frequency of the complication "reoperation" will exceed the calculated 95% CI upper limit (Table 3). This will be assessed after every 10 patients in the treatment group.

11.4.7 Software program

All statistical analyses will be computed with R version 3.6.1 [43]. The R-package "binom 3.6.0" using the "*binom.test*" function (exact binomial test). Rounding to the concrete case numbers was performed with an own rounding function implementing the "round half up" method, not the round to even method usually used from the R round-function.

12 DOCUMENTATION AND DATA MANAGEMENT

12.1 Documentation of study results

A subject screening and identification Log will be completed for all enrolled subjects with the reasons for exclusion.

12.1.1 Electronic case report form (eCRF)

A web-based eCRF, based on the CLinspire software package developed by Datamedrix GmbH, will be used for this study.

For each subject enrolled, regardless of study drug initiation, an eCRF must be completed and signed by the Investigator or a designated sub-Investigator. This also applies to those subjects who fail to complete the study. If a subject withdraws from the study, the reason must be noted on the eCRF. Case report forms are to be completed on an ongoing basis.

eCRF entries and corrections will only be performed by study site staff, authorized by the Investigator. The entries will be checked by trained personnel (Monitor) and any errors or inconsistencies will be checked immediately.

Data management for the eCRFs will be performed by the Clinical Trials Coordination Centre of the Medical University of Vienna.

12.1.2 Data collection

Data collected at all visits are entered into an interactive form. The eCRFs will be source documents verified following guidelines established before study onset as detailed in the Monitoring Plan. Maintenance of the study database will be performed by the Clinical Trials Coordination Center of the Medical University of Vienna.

12.2 Safekeeping

The Investigator will maintain adequate and accurate records to enable the conduct of the study to be fully documented and the study data to be subsequently verified (according to ICH-GCP “essential documents”). These documents will be classified into two different categories: Investigator's study site file (ISF) with all essential documents regarding the study conduct, and subject clinical source documents.

The Investigator's file will contain all essential documents listed in ICH-GCP Guidelines section 8.

Subject clinical source documents include all patient hospital clinical records in original version, such as original laboratory reports, ECG, X-ray prints and other reports.

These two categories of documents must be kept on file by the Investigator for as long as needed to comply with the regulatory requirements.

12.3 Quality control and quality assurance

12.3.1 Periodic Monitoring

According to GCP at least 3 monitoring visits are scheduled. An initiation visit, one routine visit and a close out visit after the last patient has finished the study or data base lock.

The designated monitor will contact and visit the Investigator on a regularly basis and will be allowed to have direct access to all source documents needed to verify the entries in the CRFs and other protocol-related documents provided that subject confidentiality is maintained in agreement with local regulations. It will be the monitor's responsibility to inspect the CRFs at regular intervals according to the monitoring plan throughout the study, to verify the adherence to the protocol and the completeness, consistency and accuracy of the data being entered on them.

Monitoring will be performed by the Clinical Trials Coordination Centre of the Medical University of Vienna.

12.3.2 Audit and inspections

Upon request, the Investigator will make all study-related source data and records available to a qualified quality assurance auditor mandated by the sponsor or to competent authority inspectors. The main purposes of an audit or inspection are to confirm that the rights and welfare of the subjects have been adequately protected, and that all data relevant for assessment of safety and efficacy of the investigational product have appropriately been reported to the sponsor.

12.4 Reporting and publication

12.4.1 Publication of study results

The findings of this study will be published by the sponsor (Investigators) in a scientific journal and presented at scientific meetings. The manuscript will be circulated to all co-Investigators before submission. Confidentiality of subjects in reports/publications will be guaranteed.

13 ETHICAL AND LEGAL ASPECTS

13.1 Informed consent of subjects

Following comprehensive instruction regarding the nature, significance, impact and risks of this clinical trial, the patient must give written consent to participation in the study.

During the instruction the trial participants are to be made aware of the fact that they can withdraw their consent – without giving reasons – at any time without their further medical care being influenced in any way.

In addition to the comprehensive instructions given to the trial participants by the Investigator, the trial participants also receive a written patient information sheet in comprehensible language, explaining the nature and purpose of the study and its progress.

The patients must agree to the possibility of study-related data being passed on to relevant authorities.

The patients must be informed in detail of their obligations in relation to the trial participants insurance in order not to jeopardize insurance cover.

13.2 Acknowledgement / approval of the study

The Investigator (or a designated CRO) will submit this protocol and any related document provided to the subject (such as subject information used to obtain informed consent) to an Ethics Committee (EC) or Institutional Review Board (IRB). Approval from the committee must be obtained before starting the study.

The clinical trial shall be performed in full compliance with the legal regulations according to the Drug Law (AMG - Arzneimittelgesetz) of the Republic of Austria.

An application must also be submitted to the Austrian Competent Authorities (Bundesamt für Sicherheit im Gesundheitswesen (BASG) represented by the Agency for Health and Food Safety (AGES Medizinmarktaufsicht) and registered to the European Clinical Trial Database (EudraCT) using the required forms. The timelines for (silent) approval set by national law must be followed before starting the study.

13.2.1 Changes in the conduct of the study

Protocol amendments

Proposed amendments must be submitted to the appropriate CA and ECs. Substantial amendments may be implemented only after CA/EC approval has been obtained. Amendments that are intended to eliminate an apparent immediate hazard to subjects may be implemented prior to receiving CA/EC approval. However, in this case, approval must be obtained as soon as possible after implementation.

Study Termination

If the sponsor or the Investigator decides to terminate the study before the planned completion, they will notify each other in writing stating the reasons of early termination. Both the sponsor and the investigator will ensure the protection of the subjects' wellbeing. The sponsor will notify the regulatory

authority as well as the ethics committee about the premature termination. Documentation will be filed in the Trial Master File as well as in the Investigator Site File.

Clinical Study Report (CSR)

Within one year after the final completion of the study, a full CSR will be prepared by the sponsor and submitted to the EC and the competent authority.

The Investigator will be asked to review and sign the final study report.

13.3 Insurance

During their participation in the clinical trial the patients will be insured as defined by legal requirements. The Investigator of the clinical trial will receive a copy of the insurance conditions of the patient’s insurance. The sponsor is providing insurance in order to indemnify (legal and financial coverage) the Investigator/center against claims arising from the study, except for claims that arise from malpractice and/or negligence. The compensation of the subject in the event of study-related injuries will comply with the applicable regulations.

Details on the existing patient’s insurance are given in the patient information sheet.

13.4 Confidentiality

The information contained in this document, especially unpublished data, is the property of the sponsor. It is therefore provided to you in confidence as an Investigator, potential Investigator, or consultant, for review by you, your staff, and an Ethics Committee or Institutional Review Board. It is understood that this information will not be disclosed to others without written authorization from the sponsor.

13.5 Ethics and good clinical practice (GCP)

The Investigator will ensure that this study is conducted in full conformance with the principles of the "Declaration of Helsinki" (as amended at the 64th WMA General Assembly, Fortaleza, Brazil, 2013) and with the laws and regulations of the country in which the clinical research is conducted.

The Investigator of the clinical trial shall guarantee that only appropriately trained personnel will be involved in the study. All studies must follow the ICH GCP Guidelines and the regulatory requirements.

Therefore, this study follows the EU Directive embedded in the Austrian drug act.

14 REFERENCES

[1] J. Erlandsson *et al.*, "Optimal fractionation of preoperative radiotherapy and timing to surgery for rectal cancer (Stockholm III): a multicentre, randomised, non-blinded, phase 3, non-inferiority trial," *Lancet Oncol.*, vol. 18, no. 3, pp. 336–346, 2017, doi: 10.1016/S1470-2045(17)30086-4.

[2] J. Ferlay *et al.*, "Cancer incidence and mortality patterns in Europe: Estimates for 40 countries in 2012," *Eur. J. Cancer*, vol. 49, no. 6, pp. 1374–1403, 2013, doi: 10.1016/j.ejca.2012.12.027.

[3] R. Glynne-Jones *et al.*, "Rectal cancer: ESMO Clinical Practice Guidelines for diagnosis, treatment and follow-up," *Ann. Oncol.*, vol. 28, no. suppl_4, pp. iv22–iv40, Jul. 2017, doi: 10.1093/annonc/mdx224.

[4] K. M. Mahoney, P. D. Rennert, and G. J. Freeman, "Combination cancer immunotherapy and new immunomodulatory targets," *Nat. Rev. Drug Discov.*, vol. 14, no. 8, pp. 561–584, 2015, doi: 10.1038/nrd4591.

[5] J. Galon and D. Bruni, "Approaches to treat immune hot, altered and cold tumours with combination immunotherapies," *Nat. Rev. Drug Discov.*, vol. 18, no. March, pp. 10–13, 2019, doi: 10.1038/s41573-018-0007-y.

[6] D. T. Le *et al.*, "PD-1 Blockade in Tumors with Mismatch-Repair Deficiency," *N. Engl. J. Med.*, vol. 372, no. 26, pp. 2509–2520, 2015, doi: 10.1056/NEJMoa1500596.

[7] F. Pagès *et al.*, "International validation of the consensus Immunoscore for the classification of colon cancer: a prognostic and accuracy study," *Lancet*, vol. 391, no. 10135, pp. 1–12, May 2018, doi: 10.1016/S0140-6736(18)30789-X.

[8] D. T. Le *et al.*, "Mismatch repair deficiency predicts response of solid tumors to PD-1 blockade," *Science (80-.).*, vol. 357, no. 6349, pp. 409–413, Jul. 2017, doi: 10.1126/science.aan6733.

[9] M. J. Overman *et al.*, "Nivolumab in patients with metastatic DNA mismatch repair-deficient or microsatellite instability-high colorectal cancer (CheckMate 142): an open-label, multicentre, phase 2 study," *Lancet Oncol.*, vol. 18, no. 9, pp. 1182–1191, Sep. 2017, doi: 10.1016/S1470-2045(17)30422-9.

[10] L. Galluzzi, T. A. Chan, G. Kroemer, J. D. Wolchok, and A. López-Soto, "The hallmarks of successful anticancer immunotherapy.," *Sci. Transl. Med.*, vol. 10, no. 459, p. eaat7807, Sep. 2018, doi: 10.1126/scitranslmed.aat7807.

[11] W. L. Hwang, L. R. G. Pike, T. J. Royce, B. A. Mahal, and J. S. Loeffler, "Safety of combining radiotherapy with immune-checkpoint inhibition," *Nat. Rev. Clin. Oncol.*, vol. 15, no. 8, pp. 477–494, 2018, doi: 10.1038/s41571-018-0046-7.

[12] S. Demaria, C. N. Coleman, and S. C. Formenti, "Radiotherapy: Changing the Game in Immunotherapy," *Trends in Cancer*, vol. 2, no. 6, pp. 286–294, Jun. 2016, doi: 10.1016/j.trecan.2016.05.002.

[13] L. Deng *et al.*, "Irradiation and anti – PD-L1 treatment synergistically promote antitumor immunity in mice," *J. Clin. Invest.*, vol. 124, no. 2, pp. 687–695, 2014, doi: 10.1172/JCI67313.might.

[14] M. Hecht *et al.*, "PD-L1 is upregulated by radiochemotherapy in rectal adenocarcinoma patients and associated with a favourable prognosis.," *Eur. J. Cancer*, vol. 65, pp. 52–60, 2016, doi: 10.1016/j.ejca.2016.06.015.

[15] A. B. Sharabi *et al.*, "Stereotactic Radiation Therapy Augments Antigen-Specific PD-1-Mediated Antitumor Immune Responses via Cross-Presentation of Tumor Antigen.," *Cancer Immunol. Res.*, vol. 3, no. 4, pp. 345–55, Apr. 2015, doi: 10.1158/2326-6066.CIR-14-0196.

[16] S. C. Formenti *et al.*, "Radiotherapy induces responses of lung cancer to CTLA-4 blockade," *Nat. Med.*, vol. 24, no. 12, pp. 1845–1851, Dec. 2018, doi: 10.1038/s41591-018-0232-2.

[17] L. Gandhi *et al.*, "Pembrolizumab plus Chemotherapy in Metastatic Non-Small-Cell Lung Cancer.," *N. Engl. J. Med.*, vol. 378, no. 22, pp. 2078–2092, May 2018, doi: 10.1056/NEJMoa1801005.

[18] J. D. Wolchok *et al.*, "Overall Survival with Combined Nivolumab and Ipilimumab in Advanced Melanoma.," *N. Engl. J. Med.*, vol. 377, no. 14, pp. 1345–1356, 2017, doi: 10.1056/NEJMoa1709684.

[19] M. D. Hellmann *et al.*, "Nivolumab plus Ipilimumab in Lung Cancer with a High Tumor Mutational Burden," *N. Engl. J. Med.*, vol. 378, no. 22, pp. 2093–2104, 2018, doi: 10.1056/NEJMoa1801946.

[20] Y. Y. Janjigian *et al.*, "CheckMate-032 Study: Efficacy and Safety of Nivolumab and Nivolumab Plus Ipilimumab in Patients With Metastatic Esophagogastric Cancer.," *J. Clin. Oncol.*, vol. 36, no. 28,

pp. 2836–2844, Oct. 2018, doi: 10.1200/JCO.2017.76.6212.

[21] A. C. Huang *et al.*, "A single dose of neoadjuvant PD-1 blockade predicts clinical outcomes in resectable melanoma," *Nat. Med.*, vol. 25, no. March, 2019, doi: 10.1038/s41591-019-0357-y.

[22] A. Necchi *et al.*, "Pembrolizumab as Neoadjuvant Therapy Before Radical Cystectomy in Patients With Muscle-Invasive Urothelial Bladder Carcinoma (PURE-01): An Open-Label, Single-Arm, Phase II Study," *J. Clin. Oncol.*, vol. 36, no. 34, pp. 3353–3360, Dec. 2018, doi: 10.1200/JCO.18.01148.

[23] P. M. Forde *et al.*, "Neoadjuvant PD-1 Blockade in Resectable Lung Cancer," *N. Engl. J. Med.*, vol. 378, no. 21, pp. 1976–1986, May 2018, doi: 10.1056/NEJMoa1716078.

[24] T. F. Cloughesy *et al.*, "Neoadjuvant anti-PD-1 immunotherapy promotes a survival benefit with intratumoral and systemic immune responses in recurrent glioblastoma," *Nat. Med.*, vol. 25, no. 3, pp. 477–486, Mar. 2019, doi: 10.1038/s41591-018-0337-7.

[25] K. A. Schalper *et al.*, "Neoadjuvant nivolumab modifies the tumor immune microenvironment in resectable glioblastoma," *Nat. Med.*, vol. 25, no. 3, pp. 470–476, Mar. 2019, doi: 10.1038/s41591-018-0339-5.

[26] D. Dindo, N. Demartines, and P.-A. Clavien, "Classification of Surgical Complications," *Ann. Surg.*, vol. 240, no. 2, pp. 205–213, Aug. 2004, doi: 10.1097/01.sla.0000133083.54934.ae.

[27] National Cancer Institute, "Common Terminology Criteria for Adverse Events (CTCAE)." https://ctep.cancer.gov/protocolDevelopment/electronic_applications/ctc.htm#ctc_50 (accessed Dec. 19, 2019).

[28] U. B. Patel *et al.*, "Magnetic Resonance Imaging–Detected Tumor Response for Locally Advanced Rectal Cancer Predicts Survival Outcomes: MERCURY Experience," *J. Clin. Oncol.*, vol. 29, no. 28, pp. 3753–3760, Oct. 2011, doi: 10.1200/JCO.2011.34.9068.

[29] A. M. Mandard *et al.*, "Pathologic assessment of tumor regression after preoperative chemoradiotherapy of esophageal carcinoma. Clinicopathologic correlations.," *Cancer*, vol. 73, no. 11, pp. 2680–6, Jun. 1994, Accessed: May 21, 2017. [Online]. Available: http://www.ncbi.nlm.nih.gov/pubmed/8194005.

[30] A. G. Mace, R. K. Pai, L. Stocchi, and M. F. Kalady, "American joint committee on cancer and college of American pathologists regression grade: A new prognostic factor in rectal cancer," *Dis. Colon Rectum*, vol. 58, no. 1, pp. 32–44, 2015, doi: 10.1097/DCR.0000000000000266.

[31] R. Ryan *et al.*, "Pathological response following long-course neoadjuvant chemoradiotherapy for locally advanced rectal cancer," *Histopathology*, vol. 47, no. 2, pp. 141–146, 2005, doi: 10.1111/j.1365-2559.2005.02176.x.

[32] O. Dworak, L. Keilholz, and A. Hoffmann, "Pathological features of rectal cancer after preoperative radiochemotherapy.," *Int. J. Colorectal Dis.*, vol. 12, no. 1, pp. 19–23, 1997, [Online]. Available: http://www.ncbi.nlm.nih.gov/pubmed/9112145.

[33] J. D. Brierley, M. K. Gospodarowicz, and C. Wittekind, *TNM Classification of Malignant Tumours, 8th Edition*. John Wiley & Sons, Inc., 2017.

[34] R. J. Heald, E. M. Husband, and R. D. H. Ryall, "The mesorectum in rectal cancer surgery-the clue to pelvic recurrence?," *Br. J. Surg.*, vol. 69, no. 10, pp. 613–616, Oct. 1982, doi: 10.1002/bjs.1800691019.

[35] M. Stoeckius *et al.*, "Simultaneous epitope and transcriptome measurement in single cells," *Nat. Methods*, vol. 14, no. 9, pp. 865–868, Sep. 2017, doi: 10.1038/nmeth.4380.

[36] V. M. Peterson *et al.*, "Multiplexed quantification of proteins and transcripts in single cells," *Nat. Biotechnol.*, vol. 35, no. 10, pp. 936–939, Oct. 2017, doi: 10.1038/nbt.3973.

[37] J. Klughammer *et al.*, "Differential DNA Methylation Analysis without a Reference Genome.," *Cell Rep.*, vol. 13, no. 11, pp. 2621–2633, Dec. 2015, doi: 10.1016/j.celrep.2015.11.024.

[38] M. Fanelli *et al.*, "Pathology tissue-chromatin immunoprecipitation, coupled with high-throughput sequencing, allows the epigenetic profiling of patient samples.," *Proc. Natl. Acad. Sci. U. S. A.*, vol. 107, no. 50, pp. 21535–40, Dec. 2010, doi: 10.1073/pnas.1007647107.

[39] M. Fanelli, S. Amatori, I. Barozzi, and S. Minucci, "Chromatin immunoprecipitation and high-throughput sequencing from paraffin-embedded pathology tissue," *Nat. Protoc.*, vol. 6, no. 12, pp. 1905–1919, Nov. 2011, doi: 10.1038/nprot.2011.406.

[40] P. Cejas *et al.*, "Chromatin immunoprecipitation from fixed clinical tissues reveals tumor-specific enhancer profiles.," *Nat. Med.*, vol. 22, no. 6, pp. 685–91, Jun. 2016, doi: 10.1038/nm.4085.

[41] L. R. Olsen, M. D. Leipold, C. B. Pedersen, and H. T. Maecker, "The anatomy of single cell mass

cytometry data," *Cytom. Part A*, vol. 95, no. 2, pp. 156–172, Feb. 2019, doi: 10.1002/cyto.a.23621.

[42] Y. Benjamini and Y. Hochberg, "Controlling the false discovery rate: a practical and powerful approach to multiple testing," *Journal of the Royal Statistical Society*, vol. 57, no. 1. WileyRoyal Statistical Society, pp. 289–300, 1995, doi: 10.2307/2346101.

[43] R Core Team, "R: A language and environment for statistical computing." R Foundation for Statistical Computing, Vienna, Austria, 2021, [Online]. Available: https://www.r-project.org/.
