# Supplementary material for: Dual Immune Checkpoint Inhibition Plus Neoadjuvant Chemoradiotherapy in Rectal Cancer: A Randomized Clinical Trial
Source: JAMA Netw Open. 2025 Aug 22;8(8):e2527769. doi: 10.1001/jamanetworkopen.2025.27769 (PMC12374221; doi:10.1001/jamanetworkopen.2025.27769)
Supplement: Supplement 2. — eTable 1. Surgical Morbidity eTable 2. Summary of Adverse Events (Treatment-Related or Not) eTable 3. Therapy Response eTable 4. Pathological Characteristics eFigure 1. Study Design eFigure 2. Consort Flow Diagram eFigure 3. Distribution of AEs Occurring in at Least 10% of All Patients eFigure 4. Distribution of AE Grades ≥3 [file jamanetwopen-e2527769-s002.pdf]

Supplementary Online Content

Laengle J, Kuehrer I, Kulu A, et al. Dual immune checkpoint inhibition plus neoadjuvant chemoradiotherapy in rectal cancer: a randomized clinical trial. *JAMA Netw Open.* 2025;8(8):e2527769. doi:10.1001/jamanetworkopen.2025.27769

eTable 1. Surgical Morbidity

eTable 2. Summary of Adverse Events (Treatment-Related or Not)

eTable 3. Therapy Response

eTable 4. Pathological Characteristics

eFigure 1. Study Design

eFigure 2. Consort Flow Diagram

eFigure 3. Distribution of AEs Occurring in at Least 10% of All Patients

eFigure 4. Distribution of AE Grades ≥3

This supplementary material has been provided by the authors to give readers additional information about their work.

© 2025 Laengle J et al. *JAMA Network Open*.

eTable 1. Surgical Morbidity

	CRT	CRT+IPI/NIVO
**Surgical complications (Clavien-Dindo), No. (%)**		
Any Grade	20 (78)	33 (77)
Grade I	18 (69)	30 (70)
Grade II	15 (58)	14 (33)
Grade IIIa	2 (8)	2 (5)
Rectal anastomotic leak	1 (4)	1 (2)
Percutaneous drainage	1 (4)	–
Cardiac catheterization	–	1 (2)
Grade IIIb	2 (8)	2 (5)
Rectal anastomotic leak	–	1 (2)
Fascial dehiscence	1 (4)	–
Mechanical Ileus	1 (4)	1 (2)
Grade V	–	1 (2)
**Reoperation, No. (%)**	2 (8)	3 (7)
**90-day mortality, No. (%)**	–	1 (2)

eTable 2. Summary of Adverse Events (Treatment-Related or Not)

	CRT	CRT+IPI/NIVO
**ITT population, No.**	30	50
**Adverse event, No (%)**		
Any grade	30 (100)	50 (100)
≥Grade 3	8 (27)	15 (30)
Serious (SAE)	2 (7)	7 (14)[a]

[a]Out of these, 3 (6%) IPI/NIVO related.

eTable 3. Therapy Response

	CRT	CRT+IPI/NIVO
**ITT population, No.**	**30**	**50**
**Complete response (cCR or pCR), No (%)**		
Yes	9 (30)	11 (22)
No	21 (70)	39 (78)
**Major clinical response (MCR), No (%)**[a]		
MCR	18 (60)	34 (71)
Non-MCR	12 (40)	14 (29)
**Major pathological response (MPR), No (%)**[b]		
MPR	10 (38)	16 (37)
Non-MPR	16 (62)	27 (63)

© 2025 Laengle J et al. *JAMA Network Open*.

**Neoadjuvant rectal (NAR) score, No (%)**[b]		
Low	9 (35)	11 (26)
Intermediate	9 (35)	23 (53)
High	8 (30)	9 (21)

[a]Two patients in der treatment arm could not be asses for mrTRG (poor ECOG status due to trauma and 1 had claustrophobia for MRI, only PET CT available), [b]Surgery population, [§]Too less viable tissue left or pCR. *Positive lymph node at the lateral pelvic wall. [#]Liver and lung metastases at restaging, patient wish to proceed with surgery of her primary tumor. MPR: major pathological response; pCR: pathological complete response; cCR: clinical complete response; MCR: major clinical response

eTable 4. Pathological Characteristics

	CRT	CRT+IPI/NIVO
**ITT population, No.**	**30**	**50**
**Tumor differentiation, No. (%)**[b]		
Well (G1)	2 (8)	8 (19)
Moderate (G2)	21 (80)	31 (72)
Poor (G3)	2 (8)	4 (9)
Missing	1 (4)	–
**ypT stage, No. (%)**[b]		
ypT0	6 (23)	7 (16)
ypT1	1 (4)	6 (14)
ypT2	9 (35)	14 (33)
ypT3	7 (27)	15 (35)
ypT4	3 (12)	1 (2)
**ypN stage, No. (%)**[b]		
ypN0	18 (70)	31 (72)
ypN1a	2 (8)	7 (16)
ypN1b	2 (8)	2 (5)
ypN1c	–	1 (2)
ypN2b	4 (15)	2 (5)
**ypM stage, No. (%)**[b]		
ypM0	24 (92)	43 (100)
ypM1a*	1 (4)	–
ypM1b[#]	1 (4)	–
**Lymphatic invasion, No. (%)**[b]		
L0	24 (92)	39 (91)
L1	2 (8)	4 (9)

© 2025 Laengle J et al. *JAMA Network Open*.

**Vascular invasion, No. (%)**[b]		
V0	23 (89)	43 (100)
V1	3 (11)	–
**Perineural invasion, No. (%)**[b]		
Pn0	21 (84)	39 (91)
Pn1	4 (16)	4 (9)
Missing	1	–
**Residual tumor classification, No. (%)**[b]		
R0	26 (100)	43 (100)
**UICC TNM stage (8th edition), No. (%)**[b]		
Stage 0	6 (23)	7 (16)
Stage I	9 (35)	13 (30)
Stage IIA	3 (11)	10 (23)
Stage IIC	–	1 (2)
Stage IIIA	1 (4)	6 (14)
Stage IIIB	–	5 (12)
Stage IIIC	5 (19)	1 (2)
Stage IVA	1 (4)	–
Stage IVB	1 (4)	–
**Mismatch repair (MMR) status, No. (%)**[b]		
Proficient (pMMR)	23 (88)	42 (98)
Deficient (dMMR)	1 (4)	–
Missing[§]	2 (8)	1 (2)
***KRAS* status, No. (%)**[b]		
Wilde type	9 (30)	16 (37)
Mutated	10 (33)	13 (30)
Missing[§]	7 (27)	14 (33)
***BRAF* status, No. (%)**[b]		
Wilde type	19 (73)	29 (67)
Mutated	0 (0)	0 (0)
Missing[§]	7 (27)	14 (33)
***NRAS* status, No. (%)**[b]		
Wilde type	18 (69)	27 (63)
Mutated	1 (4)	2 (5)
Missing[§]	7 (27)	14 (32)
***PTEN* status, No. (%)**[b]		
Wilde type	17 (65)	27 (63)
Mutated	1 (4)	0 (0)
Missing[§]	8 (31)	16 (37)
***PIK3CA* status, No. (%)**[b]		
Wilde type	17 (65)	25 (58)
Mutated	2 (8)	2 (5)
Missing[§]	7 (27)	16 (37)
***TP53* status, No. (%)**[b]		

© 2025 Laengle J et al. *JAMA Network Open*.

Wilde type	9 (35)	12 (28)
Mutated	9 (35)	15 (35)
Missing[§]	8 (30)	16 (37)

[a]Two patients in the treatment arm could not be assessed for mrTRG (poor ECOG status due to trauma and 1 had claustrophobia for MRI, only PET CT available). [b]Surgery population. [§]Too less viable tissue left or pCR. *Positive lymph node at the lateral pelvic wall. [#]Liver and lung metastases at restaging, patient wish to proceed with surgery of her primary tumor. MPR: major pathological response; pCR: pathological complete response; cCR: clinical complete response; MCR: major clinical response

© 2025 Laengle J et al. *JAMA Network Open*.

eFigure 1. Study Design

Graphical representation of the prospective, randomized, open-label, multicenter, phase II investigator-initiated trial (IIT) design.

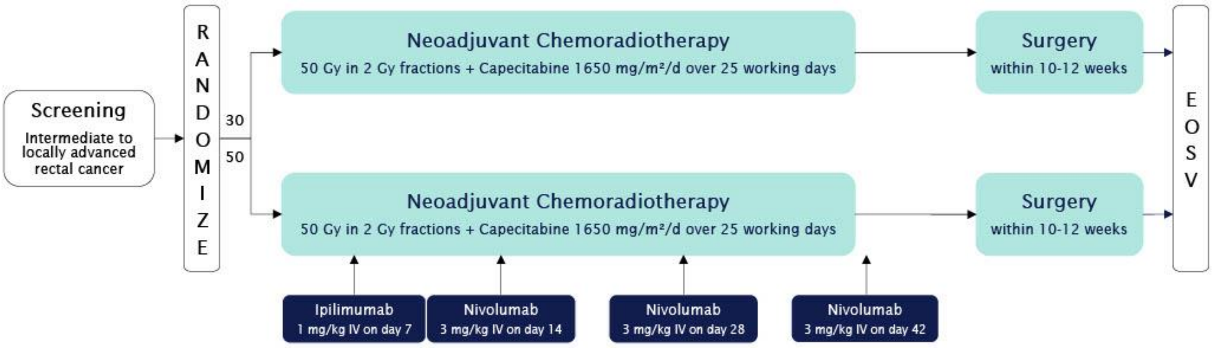

eFigure 2. Consort Flow Diagram

Graphical illustration of patient disposition during screening, neoadjuvant treatment, and curative resection. CRT, chemoradiotherapy; IPI, ipilimumab; NIVO, nivolumab; ECOG, Eastern Cooperative Oncology Group.

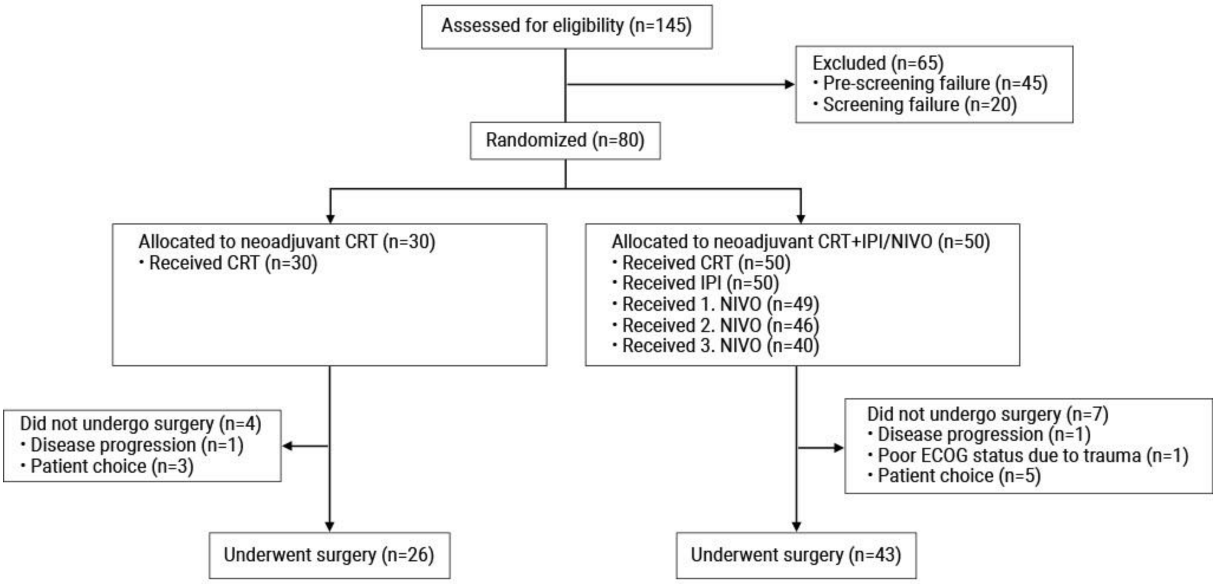

© 2025 Laengle J et al. *JAMA Network Open*.

eFigure 3. Distribution of AEs Occurring in at Least 10% of All Patients

Turquoise indicates the IPI/NIVO arm and red specifies the chemoradiotherapy (CRT) arm.

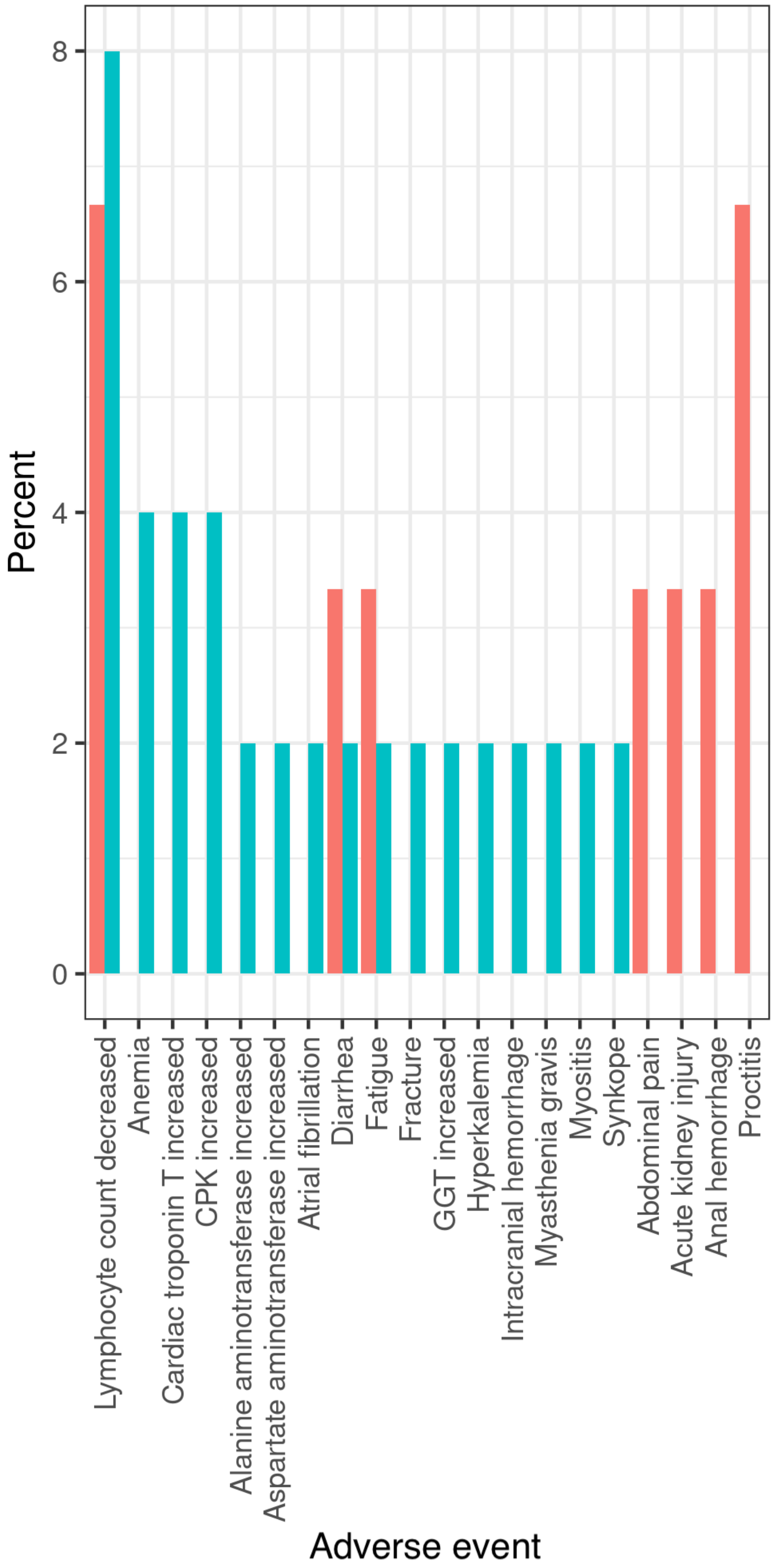

© 2025 Laengle J et al. *JAMA Network Open*.

eFigure 4. Distribution of AE Grades ≥3

Turquoise indicates the IPI/NIVO arm and red specifies the chemoradiotherapy (CRT) arm

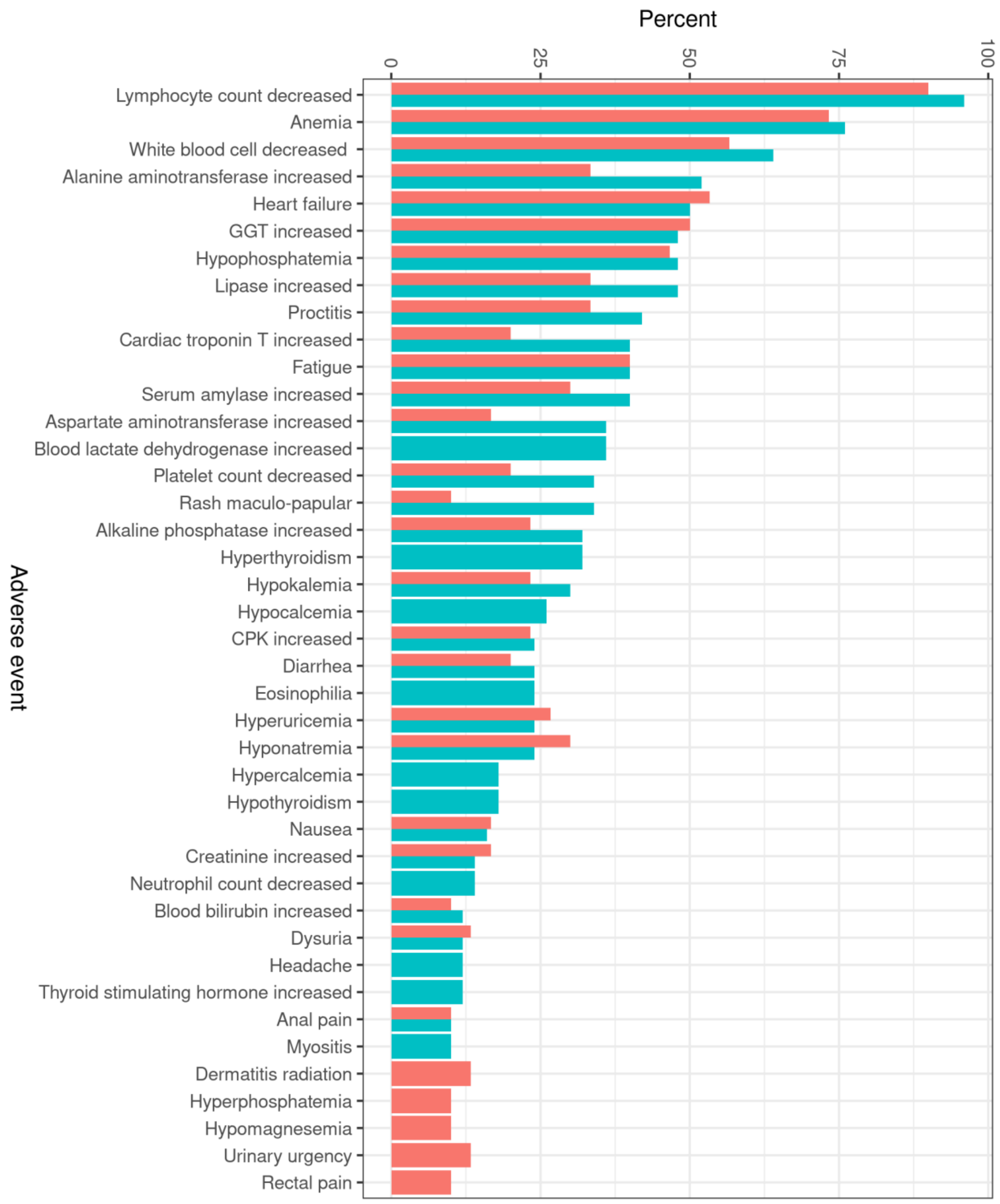

© 2025 Laengle J et al. *JAMA Network Open*.
