# Supplementary material for: Dual Immune Checkpoint Inhibition Plus Neoadjuvant Chemoradiotherapy in Rectal Cancer: A Randomized Clinical Trial
Source: JAMA Netw Open. 2025 Aug 22;8(8):e2527769. doi: 10.1001/jamanetworkopen.2025.27769 (PMC12374221; doi:10.1001/jamanetworkopen.2025.27769)
Supplement: Supplement 3. — Data Sharing Statement [file jamanetwopen-e2527769-s003.pdf]

Data Sharing Statement

Laengle. Dual Immune Checkpoint Inhibition Plus Neoadjuvant Chemoradiotherapy in Rectal Cancer. *JAMA Netw Open*. Published August 22, 2025.
doi:10.1001/jamanetworkopen.2025.27769

Data
Additional Information: ClinicalTrials.gov https://clinicaltrials.gov/ NCT04124601
Data available: Yes
Data types: Deidentified participant data
How to access data: Anonymized data will be made available upon reasonable academic request. Protected health information will not be shared. Interested researchers should contact the corresponding author, providing approval from their local Institutional Review Board (IRB) and a signed academic non-disclosure agreement (NDA).
When available: With publication

Supporting Documents
Document types: None

Additional Information
Who can access the data: De-identified participant data will be made available exclusively to researchers whose proposed use of the data has been formally reviewed and approved. Data sharing will be restricted to analyses specifically outlined in approved research proposals. No personally identifiable information or PHI will be shared. Interested researchers should contact the corresponding author and must provide proof of approval from their local IRB, along with a signed academic NDA.
Types of analyses: The data will be made available exclusively for the specified analyses outlined in the approved research proposal, and not for any other purposes.
Mechanisms of data availability: The requested data will be made available after approval of the proposal and upon receipt of a signed data access agreement.
